# The effect of iron therapy on oxidative stress and intestinal microbiota in inflammatory bowel diseases: A review on the conundrum

**DOI:** 10.1016/j.redox.2023.102950

**Published:** 2023-10-30

**Authors:** R. Loveikyte, A.R. Bourgonje, H. van Goor, G. Dijkstra, A.E. van der Meulen – de Jong

**Affiliations:** a Department of Gastroenterology and Hepatology, Leiden University Medical Center, Leiden, the Netherlands; b Department of Gastroenterology and Hepatology, University Medical Center Groningen, University of Groningen, Groningen, the Netherlands; c The Henry D. Janowitz Division of Gastroenterology, Department of Medicine, Icahn School of Medicine at Mount Sinai, New York, NY, United States; d Department of Pathology and Medical Biology, University Medical Center Groningen, University of Groningen, Groningen, the Netherlands

**Keywords:** Iron deficiency, Oxidative stress, Intestinal microbiota, Iron supplementation, Inflammatory bowel disease

## Abstract

One in five patients with Inflammatory Bowel Disease (IBD) suffers from anemia, most frequently caused by iron deficiency. Anemia and iron deficiency are associated with worse disease outcomes, reduced quality of life, decreased economic participation, and increased healthcare costs. International guidelines and consensus-based recommendations have emphasized the importance of treating anemia and iron deficiency. In this review, we draw attention to the rarely discussed effects of iron deficiency and iron therapy on the redox status, the intestinal microbiota, and the potential interplay between them, focusing on the clinical implications for patients with IBD. Current data are scarce, inconsistent, and do not provide definitive answers. Nevertheless, it is imperative to rule out infections and discern iron deficiency anemia from other types of anemia to prevent untargeted oral or intravenous iron supplementation and potential side effects, including oxidative stress. Further research is necessary to establish the clinical significance of changes in the redox status and the intestinal microbiota following iron supplementation.

## Highlights

• Iron deficiency and anemia affect quality of life and disease outcomes in patients with inflammatory bowel disease.
• Iron deficiency alters redox status and the intestinal microbiota.
• Oral and intravenous iron, especially in excess, can alter redox status and the intestinal microbiota.

## 1 Introduction

Inflammatory Bowel Disease (IBD)—encompassing ulcerative colitis (UC) and Crohn's disease (CD)—is an immune-mediated condition that is becoming increasingly more prevalent worldwide and is marked by a relapsing-remitting gastrointestinal inflammation [1,2]. IBD can also manifest outside the gastrointestinal tract, with anemia being the most common systemic manifestation in approximately one in five patients [3,4]. The prevalence of anemia is higher in patients with newly diagnosed or active IBD due to various factors such as inflammation- or medication-induced myelosuppression, chronic bleeding, or nutritional deficiencies caused by malabsorption or poor intake [4,5]. Despite a plethora of predisposing factors, iron deficiency anemia (IDA) is the most common type of anemia in IBD [3,[6], [7], [8]].

Iron is essential for most physiologic functions such as energy metabolism, immune function, oxygen transport, and neurotransmitter synthesis [9]. Unsurprisingly, anemia and non-anemic iron deficiency (NAID) have been associated with worse disease outcomes, reduced quality of life (QoL), decreased economic participation, worse cognitive functioning, and increased healthcare costs [[10], [11], [12], [13], [14]]. The diminished QoL of these patients has even been likened to patients with advanced oncologic diseases [15]. Hence, the importance of regular screening and prompt treatment of anemia in patients with IBD has been emphasized in the European Crohn's and Colitis Organisation (ECCO) guidelines [5,16].

The goal of IDA treatment is to normalize hemoglobin levels and replenish iron stores. According to the ECCO guidelines, patients with quiescent or mildly active IBD should be treated with oral iron therapy in doses not exceeding 100 mg of daily elemental iron [5]. In contrast, intravenous iron is recommended as the first-line therapy in patients with active IBD or cases of severe anemia [5]. Despite the guidelines and consensus-based recommendations, studies have shown that anemia and NAID are often undertreated or even left untreated [3,4,7,17]. Both iron modalities have been shown effective in patients with IBD, but frequent side effects and the established consequences of iron overload in patients with hemochromatosis have arguably made physicians cautious about prescribing iron [[18], [19], [20], [21]]. In IBD, iron has been postulated to contribute to inflammation, oxidative stress, and intestinal dysbiosis, which might explain low prescription rates, neglecting the consequences of iron deficiency.

In this review, we draw attention to the lesser-known effects of iron therapy. We explore the effect of iron therapy on oxidative stress and then delve into the effect of iron therapy on the intestinal microbiota. Finally, we discuss the potential interplay between iron status, systemic redox equilibrium, and intestinal microbiota, focusing on the clinical implications for patients with IBD. We emphasize the importance of future research and active management of anemia and NAID to restore physiological functions while balancing against the potential side effects.

## 2 Iron metabolism

Iron is vital for the functioning of all living organisms as it can easily transition between different oxidation states and participate in various physiological processes, such as energy metabolism, immune function, oxygen transport, and neurotransmitter synthesis [9]. Under physiological conditions, the body contains 3–5 g of iron: hemoglobin contains approximately half of bodily iron, approximately 300 mg is stored in myoglobin and cytochromes, and only 3–4 mg of iron can be found in plasma bound to transferrin. The remaining iron is stored as ferritin primarily in the liver, spleen, and bone marrow [19,22]. However, too little or too much iron can be harmful, e.g., iron overload can cause oxidative stress and tissue injury, large or repeated doses of ferric carboxymaltose can upregulate Fibroblast Growth Factor 23 and lead to renal phosphate wasting and secondary hyperparathyroidism, meanwhile concurrent hypoxia and iron deficiency can lead to pulmonary hypertension [23,24]. Therefore, the body must tightly regulate its physiological iron content.

### 2.1 Systemic iron metabolism

**Fig. 1:**
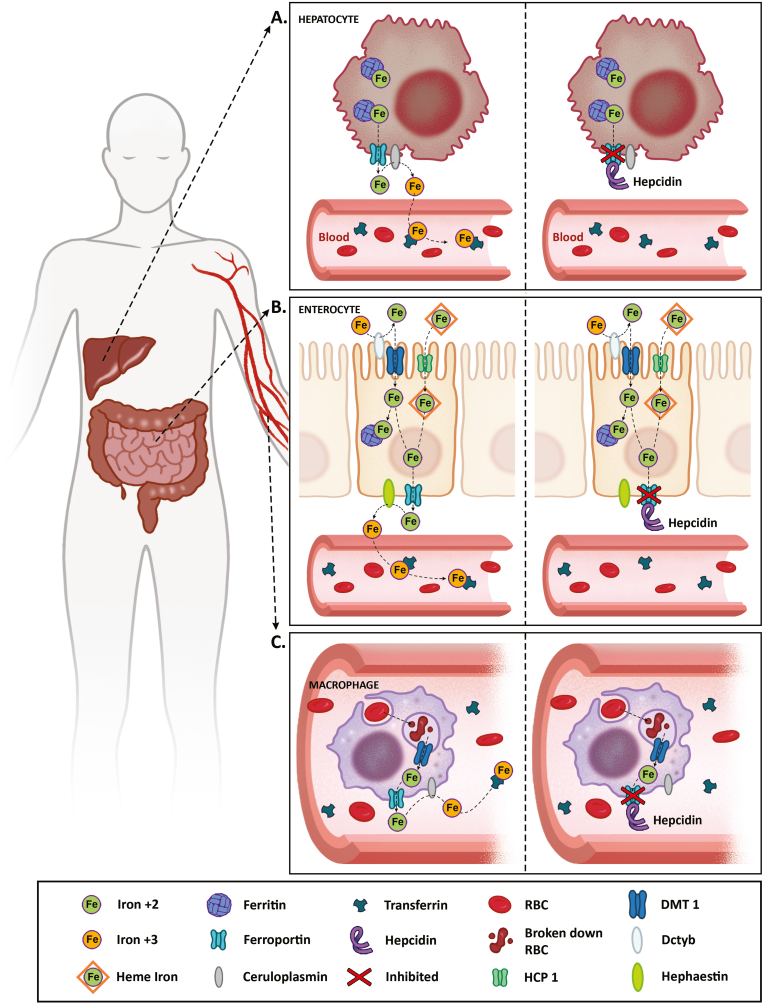
**Iron absorption and recycling**. Hepcidin plays a critical role in regulating systemic iron availability and enteral iron absorption by modulating FPN levels. During iron deficiency, stored iron in hepatocytes (A) is exported to the systemic circulation by FPN. Here ferrous iron is oxidized by ceruloplasmin and bound to transferrin for further transport and utilization. In enterocytes (B), non-heme iron is reduced from ferric to ferrous iron by Dcytb at the apical border. Ferrous iron is taken up into the enterocyte by DMT1, where it is stored bound to ferritin or exported by FPN to the systemic circulation, where it gets oxidized by hephaestin and bound to transferrin. Heme-iron uptake by enterocytes is not entirely understood, but it is believed to be absorbed by HCP1 and degraded by HO1. The released iron is bound to ferritin or exported into the systemic circulation, as necessary. Finally, senescent RBCs are phagocytosed by macrophages (C). RBCs are degraded in the lysosome, and heme is exported to the cytosol via HCP1. In the cytosol, iron is extracted by HO1 and stored as ferritin or exported to the systemic circulation by FPN. In an iron-replete state or cases of elevated hepcidin, hepcidin blocks, internalizes and degrades FPN. This causes iron restriction within the cells and prevents iron export to the circulation. DMT1: divalent metal transporter 1, Dcytb: duodenal cytochrome B, FPN: ferroportin, Fe^3+^: ferrous iron, Fe^2+^: ferric iron, HO1: heme oxygenase 1, HCP1: heme carrier protein 1, RBC: red blood cell. (For interpretation of the references to colour in this figure legend, the reader is referred to the Web version of this article.)

Most of the iron present in the body is stored either in hepatocytes or recycled from senescent red blood cells by macrophages. Additional iron is absorbed from food or iron supplements primarily in the duodenum and proximal jejunum. As depicted in Fig. 1, non-heme iron (e.g., iron from plant-based food) is reduced from Fe^3+^ to Fe^2+^ by duodenal cytochrome B (Dcytb) at the apical surface of the enterocyte and taken up by the Divalent Metal Transporter 1 (DMT1) [25]. Iron within the cell is exported to the systemic circulation by ferroportin (FPN)—the only known cellular iron exporter—at the basolateral membrane. Dietary heme iron is also absorbed by the enterocytes and exported to the systemic circulation via FPN, although its absorption at the apical side is less well understood [25].

**Fig. 2:**
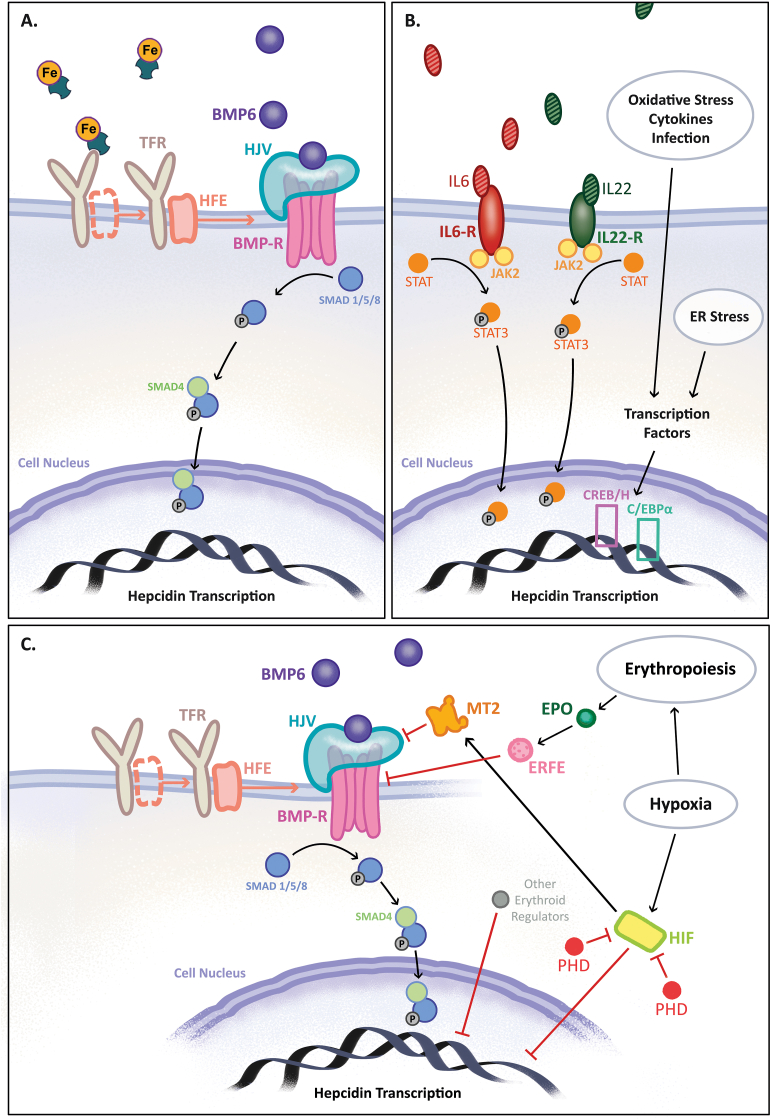
**Regulation of hepcidin. (A)** During iron overload, Tf-Fe attaches to TFR1, causing HFE to dissociate from TFR1 and bind to TFR2. The TFR-HFE complex binds to HJV, which is important for further signaling by BMP6. Elevated BMP6 levels during iron excess lead to its binding to the BMP-R and co-receptor HJV, triggering SMAD1/5/8 phosphorylation and binding to SMAD4. This complex then enters the cell nucleus and induces HAMP transcription. **(B)** During inflammation, IL-6 and IL-22 bind to their receptors, which activates JAK2 and leads to STAT3 phosphorylation. Phosphorylated STAT3 translocates to the cell nucleus and upregulates hepcidin by inducing transcription of the HAMP gene. In addition, other cytokines, oxidative stress, ER stress, and infectious inflammation upregulate hepcidin by inducing transcription factors, such as the CREBH or C/EBPα. **(C)** Iron deficiency, hypoxia, and ineffective erythropoiesis can downregulate hepcidin through multiple pathways that are not entirely understood. Under normoxic conditions, PHDs inhibit HIFs. During hypoxia, HIFs upregulate protease MT2, which inhibits BMP-SMAD signaling and decreases hepcidin expression. Hypoxia can also directly downregulate HAMP transcription. During increased erythropoiesis, EPO upregulates ERFE, which is known to inhibit BMP-SMAD signaling. Other potential erythroid regulators, such as GDF15, might also downregulate hepcidin. BMP6: bone morphogenetic protein 6, BMP-R: BMP receptor, EPO: erythropoietin, ERFE: erythroferrone, ER: endoplasmic reticulum, GDF15: growth and differentiation factor 15, HFE: human hemochromatosis protein, HJV: hemojuvelin, HIF: hypoxia-inducible transcription factor, IL: interleukin, JAK2: Janus kinase 2, MT2: matriptase 2, PHD: prolyl hydroxylase, SMAD: mothers against decapentaplegic, STAT3: signal transducer and activator of transcription-3, Tf-Fe: iron bound to transferrin, TFR: transferrin receptor.

Intestinal iron absorption and systemic iron availability are regulated by the master iron regulator: hepcidin, a protein produced mainly by the liver, which acts by modulating FPN (Fig. 2) [[26], [27], [28]]. Multiple factors influence the expression of hepcidin: (infectious) inflammation and iron overload increase hepcidin as a protective mechanism to prevent free radical production and bacterial growth [21]. Hepcidin then blocks, internalizes, and degrades FPN leading to iron restriction within iron-storing cells, such as enterocytes, macrophages, and hepatocytes. In contrast, the expression of hepcidin is suppressed during iron deficiency, ineffective erythropoiesis, or hypoxia, which stabilizes FPN expression for efficient iron export from iron-storing cells to the systemic circulation [27,28]. In turn, exported iron is oxidized by hephaestin or ceruloplasmin and bound to transferrin, an iron transport protein, to form holo-transferrin that transports iron to other tissues for further utilization [25]. Unfortunately, hepcidin and its protective role is a double-edged sword in patients with chronic inflammatory conditions, such as IBD, whereby inflammation and iron deficiency may coexist and could lead to inappropriately elevated hepcidin levels, hindering adequate response to iron therapy.

### 2.2 Intracellular iron metabolism

**Fig. 3:**
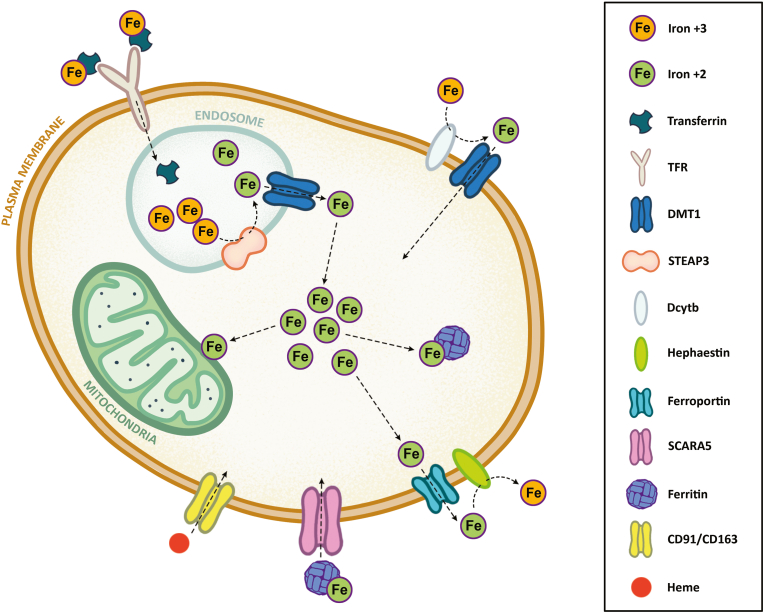
**A high-level overview of intracellular iron metabolism.** At the cellular membrane, holo-transferrin binds to TFR1, after which the holo-transferrin and TFR1 complex is internalized by endocytosis. In the endosome, iron is reduced by STEAP and exported to the cytoplasm via DMT1. The iron in the cytoplasm either enters the labile iron pool and is stored as ferritin, transported to mitochondria for further utilization, or exported through FPN in cases of cellular iron excess. Similarly, cells can also acquire iron as ferritin, transported into the cell via SCARA5, or as heme bound to haptoglobin or hemopexin, which are transported through CD91/CD163 receptors. CD91/CD163: scavenger receptors clusters of differentiation 91 and 163, DMT1: divalent metal transporter 1, Dcytb: duodenal cytochrome B, FPN: ferroportin, Fe^3+^: ferrous iron, Fe^2+^: ferric iron, HP: hephaestin, STEAP3: 6-transmembrane epithelial antigen of the prostate 3, SCARA5: scavenger receptor class A member 5, TFR1: transferrin receptor 1.

Most cells in the body import, export, or recycle iron as holo-transferrin or as hemoglobin, ferritin, and non-transferrin-bound iron (NTBI). At the cellular membrane, holo-transferrin binds to transferrin receptor 1 (TFR1), after which the holo-transferrin and TFR1 complex is internalized by endocytosis (Fig. 3). In the endosome, Fe^3+^ is reduced by the 6-transmembrane epithelial antigen of the prostate (STEAP) to Fe^2+^ and exported to the cytoplasm via DMT1. The iron in the cytoplasm either enters the labile iron pool and is stored as ferritin, transported to mitochondria for further utilization, or exported through FPN in cases of cellular iron excess [9,25].

Iron-regulating protein 1 (IRP1) and IRP2 regulate cellular iron storage and intake. IRPs bind to iron-responsive elements (IREs) located at the untranslated regions (UTRs) of the messenger RNAs (mRNAs) encoding proteins related to iron metabolism, e.g., TFR1, DMT1, FPN, hypoxia-inducible factor-1/2⍺, and ferritin [25]. In cases of iron deficiency, IRPs bind to 5′ UTR of ferritin mRNA and 3’ UTR of the TFR1 mRNA, suppressing iron storage and increasing iron uptake. The opposite happens in iron-replete cells, leading to iron storage as ferritin and limited iron uptake through TFR1 [9,25].

## 3 Oxidative stress

Reactive oxygen species (ROS)—e.g., superoxide (O_2_^−^) or hydrogen peroxide (H_2_O_2_)—are oxygen-derived molecules formed as a byproduct of physiological processes such as mitochondrial respiration. ROS are important signaling molecules for proper cellular metabolism and function, immune system, autophagy, regulation of arterial blood flow, and thyroid hormone synthesis [29,30]. Unfortunately, excess ROS can lead to extensive cellular and molecular damage, which may perpetuate inflammation and result in tissue injury [31].

Antioxidant enzymes (e.g., superoxide dismutase [SOD], catalase [CAT], and glutathione peroxidase [GPx]) and other antioxidants (e.g., protein-bound free thiols, vitamin E, β-carotene, low-molecular-weight thiols, such as glutathione [GSH] and cysteine) play an important role in neutralizing ROS. In IBD, disrupted redox signaling and oxidative stress have been associated with clinical and endoscopic disease severity, disease exacerbation, fibrosis, and increased risk of malignancy due to mutagenesis relating to accumulating DNA damage [[31], [32], [33]]. Nevertheless, disrupted redox signaling or oxidative stress does not solely refer to quantitative overproduction of ROS but may also relate to pathological dysregulation of redox-receptive regulatory enzymes or reactive species interfering with redox-regulated metabolic pathways, and shift in location, i.e., ROS formation at sites where little production would be expected under physiological conditions [34]. In IBD, inflammation is accompanied by excess production of ROS, reactive nitrogen species (RNS), and reactive sulfur species (RSS) that lead to oxidative stress, which is considered an important pathophysiological effector mechanism in IBD [32,33,35]. Hypoxia, whether related to blood loss or inflammation, is also an important driver of oxidative stress in patients with IBD. Under hypoxic conditions, the expression of HIF-1⍺ and HIF-2⍺ increases, leading to increased ROS production. The exact underlying mechanisms of hypoxia-driven oxidative stress are not well understood; however, it is postulated that HIF regulates several complexes in the electron transfer chain to maintain homeostatic mitochondrial respiration. For instance, HIF1-⍺ inhibits pyruvate dehydrogenase, leading to a decreased pyruvate and acetyl coenzyme A conversion that actively reduces mitochondrial oxygen consumption [36]. Additionally, studies have suggested that ROS itself can stabilize HIF-1⍺ that, in turn, promotes glycolysis in non-hypoxic conditions and generates more ROS, creating a vicious cycle [37]. Despite established connections between IBD, inflammation, hypoxia, and oxidative stress, the role of HIFs in iron metabolism regulation makes this interplay even more complex, emphasizing the need to carefully assess and weigh the risks and benefits of oral and intravenous iron therapy in patients with IBD.

### 3.1 Oxidative stress and iron status

One in five patients with IBD suffers from anemia, most frequently caused by iron deficiency that has also been linked to oxidative stress [3,32,38,39]. Iron is essential for the synthesis of various proteins, such as myoglobin, cytochrome proteins, ribonucleotide reductase, and mitochondrial aconitase [9]. Iron deficiency impairs the function of iron-containing antioxidants, such as catalase and GPx, which can have deleterious effects [9]. For instance, increased ROS alter the properties of erythrocyte membrane: decrease cell deformability, increase membrane rigidity, and increase echinocyte formation [40,41]. Studies have shown that erythrocytes from iron-deficient subjects undergo lysis more rapidly compared with normal erythrocytes upon *in vitro* exposure to hydrogen peroxide [40,42].

Furthermore, iron deficiency has been linked to oxidative stress resulting from mitochondrial dysfunction. A study, conducted with mice, by Walter et al. suggests that a lack of heme, decreased cytochrome activity in the electron transport chain, and the uncoupling of mitochondria can increase superoxide release during iron deficiency [43]. Concurrently, increased iron absorption in iron-deficient states increases DMT1-mediated copper absorption, which can also generate ROS through a Fenton-like reaction and impair mitochondrial function. Lastly, the authors also postulated that changes in intracellular iron trafficking during prolonged iron deficiency could contribute to increased ROS production owing to increased IRP1 activity and reduced ferritin synthesis [43,44]. While the literature on the signaling and mechanisms underlying the accumulation of oxidative stress during iron deficiency is limited, evidence of increased lipid peroxidation and decreased antioxidant defenses has also been observed in patients with IDA [45,46].

In contrast to iron deficiency, it is widely accepted that iron excess causes oxidative stress through the Fenton reaction. ROS and H_2_O_2_ can then oxidize proteins, lipids, and nucleic acids. Lipid peroxidation generates even more ROS, initiating a chain reaction that can lead to cell apoptosis and tissue injury, whereas nucleic acid peroxidation can lead to mutations and single- or double-strand breaks [25,47]. Malondialdehyde (MDA), thiobarbituric acid reactive substances (TBARS), or isoprostanes measured in the blood are the most frequently encountered biomarkers in the existing literature and reflect lipid peroxidation; 8-hydroxy-20-deoxyguanosin (8-OHdG) reflects DNA oxidation; and protein carbonyls or advanced oxidation protein products (AOPP) reflect protein peroxidation [48,49]. Scientists have used various biomarkers; however, most biomarkers represent oxidized products in tissue or blood, which often constitute downstream end-products originating from local inflammatory processes. In addition, many of these substances are also reactive and capable of inducing oxidative damage at sites distant from the inflamed tissue [32,50,51]. The difficulty quantifying oxidative stress and a lack of understanding of the actual interactions occurring *in vivo* limits the interpretation of existing literature. Despite these limitations, understanding the effect of free or excess iron from iron therapy is imperative to adequately manage iron deficiency, which itself has been associated with oxidative stress. The impact of iron therapy on oxidative stress, particularly in patients with inflammatory conditions like IBD, remains unclear; therefore, we have performed a literature search, described in Supplementary Table S1, to evaluate evidence from human studies. In the following sections, we summarize the findings.

### 3.2 Oxidative stress: oral iron

Over the past 23 years, fewer than 30 studies have investigated the effect of oral iron therapy on redox status in healthy adults and patients. An overview of the studies is presented in Supplementary Table S2. In nine healthy males, iron supplementation with 150 mg carbonyl iron and co-supplementation with 2 g vitamin C improved redox status, as indicated by decreased erythrocyte MDA levels and increased erythrocyte CAT activity [52]. In contrast, most studies did not observe changes in oxidative stress when evaluating oral iron therapy with 25–120 mg iron as ferrous fumarate or sulfate, although one study found that even a single 25 mg dose of ferrous sulfate increased ROS generation [[53], [54], [55], [56], [57]]. In healthy pregnant or lactating women, low-dose supplementation (≤36 mg daily iron) also did not affect 8-OHdG, isoprostane, or total antioxidant activity [58,59]. However, conflicting results were reported in this population for oral iron supplementation with doses exceeding 60 mg; only a few of these studies reported increases in oxidative stress, e.g., an increase in TBARS or changes in total antioxidant capacity [[60], [61], [62]].

Compared with healthy volunteers, patients with IDA had higher baseline MDA levels and lower levels of antioxidant enzymes. Oral iron therapy improved redox status in these patients, marked by a decrease in MDA and an increase in SOD and CAT [46,[63], [64], [65]]. In addition, in a small group of patients on maintenance dialysis, oral iron therapy with sucrosomial iron decreased protein carbonyls, di-tyrosines, and AOPPs compared with intravenous iron [66]. Similar findings were observed in patients with IBD: daily supplementation with 120 mg ferrous fumarate did not affect levels of plasma MDA, antioxidants, and antioxidant enzymes compared with intravenous iron [67]. Interestingly, a study in patients with IBD found that daily supplementation with 200 mg iron polymaltose did not affect MDA, antioxidants, or urine 8-isoprostaglandin-F_2α_, whereas 100 mg ferrous fumarate increased MDA concentrations [68]. In short, the available evidence in healthy volunteers and patients is inconsistent, which may stem from different iron formulations, doses, and supplementation regimens used.

### 3.3 Oxidative stress: intravenous iron

Within the last 23 years, fewer than 50 studies have been performed to investigate the effect of intravenous iron therapy on oxidative stress markers in healthy adults and patients (an overview is presented in Supplementary Table S2). In healthy volunteers, administration of 100 mg intravenous iron sucrose resulted in a four-fold increase in NTBI levels, and a transient ROS increase was detected by electron spin resonance as early as 10 min after the infusion [69]. In contrast, repeated 300 mg iron sucrose infusions in healthy pregnant women decreased serum SOD but did not affect serum MDA levels [70].

Most studies with intravenous iron were performed in patients with CKD or patients on maintenance hemodialysis and utilized 40–250 mg intravenous iron sucrose, iron gluconate, or iron dextran. Regardless of the intravenous iron formulation or the administered dose, most studies reported increased DNA, protein, or lipid peroxidation and decreased antioxidants (Supplementary Table S2). In contrast, studies showed no change in oxidative stress after high-dose (i.e., 500–1000 mg) intravenous iron therapy with third-generation formulations [[71], [72], [73], [74], [75]]. The difference in the observed impact between older and newer generations of intravenous iron formulations might be related to the amount of NTBI they generate [76,77]. In short, it remains unclear whether the increase in oxidative stress is transient and whether it affects clinical outcomes; however, the third-generation intravenous iron formulations might be safer than the older generations.

To summarize, the existing literature regarding the effect of oral and intravenous iron on oxidative stress, especially in patients with IBD, is scarce and inconsistent. The studies used different iron formulations, dosages, supplementation regimens, and methods to characterize oxidative stress. Compared with the current-day therapy, the studies have used older-generation iron formulations, higher daily oral iron doses, and did not utilize intermittent supplementation as proposed by Stoffel et al. [78,79]. Most studies also were performed with small sample sizes, which may not ensure adequate statistical power. All in all, oral iron therapy is generally associated with lower oxidative stress than intravenous iron. More research is needed to evaluate when and for how long to prescribe iron therapy to ameliorate the effects of iron deficiency while not inducing secondary injury by excess unbound iron and reactive species.

## 4 Intestinal microbiota

**Fig. 4:**
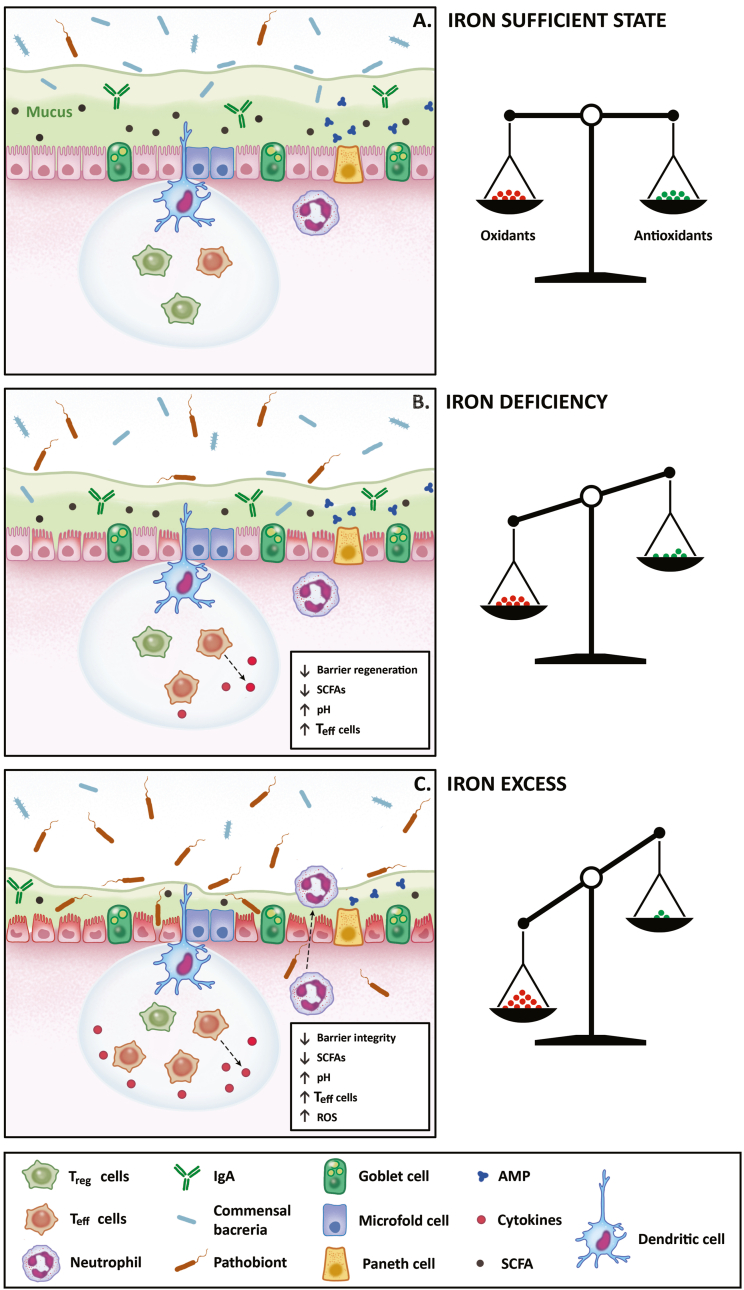
**High-level overview of changes in redox status and the intestinal microbiota during iron deficiency and excess. (A)** Under physiological conditions, the intestines are lined by a single layer of intestinal cells that do not come into direct contact with bacteria. In order to keep this physiological homeostasis, the intestines utilize several defense mechanisms. Firstly, enterocytes and goblet cells produce mucus that makes a thick inner layer, which is almost impenetrable to the bacteria, and an outer layer, which is less thick and can contain commensal bacteria. Secondly, Paneth cells and enterocytes secrete antimicrobial proteins and immunoglobulins into the mucus layer. Thirdly, the dendritic and microfold cells sample bacterial antigens and induce inflammatory cascades directed against these pathogens, which involves regulatory and effector T cells and pro- or anti-inflammatory cytokines. Finally, the intestines contain neutrophils which can trap and eliminate the bacteria in an oxidative burst. Under normal physiologic conditions, there is a balance between antioxidants and prooxidants, which prevents tissue and cell damage. **(B)** During iron deficiency, antioxidant activity is reduced and might result in oxidative stress. In the intestines, bacteria compete for iron leading to decreases in SCFA-producing bacteria, which increases luminal pH and pathobiont virulence, and results in decreased intestinal barrier regeneration. In addition, SCFAs modulate immune responses via the NF-kβ pathway, which ensures the balance between regulatory and effector T cells and anti-inflammatory cytokine production. However, during iron deficiency, the lack of SCFAs leads to increased production of effector T cells and pro-inflammatory cytokines. **(C)** During iron excess, there is an imbalance between antioxidant availability and the production of reactive species, resulting in oxidative stress. In the intestines, fewer SCFA-producing bacteria might result in changes similar to those during iron deficiency. However, pathogenic bacteria increase in abundance and virulence during iron excess and can damage the epithelium. In addition, oxidative burst by neutrophils in response to bacteria creates more reactive oxygen and nitrogen species, which further increases the damage to the intestinal epithelium. AMP: antimicrobial peptides, IgA: immunoglobulins, ROS: reactive oxygen species, SCFAs: short-chain fatty acids, Teff: T effector cells, Treg: T regulatory cells.

The intestines contain a large number of commensal bacteria that aid food digestion, nutrient absorption, regulation of the immune system, and protection against colonization by pathogenic bacteria [80]. The intestinal epithelial cells do not come into direct contact with the pathogenic bacteria under physiological conditions, and there are several defense mechanisms to prevent potential damage. Firstly, the intestinal epithelium is almost impervious to bacteria because it is lined by mucus, secreted by enterocytes and goblet cells [81] (Fig. 4A). [82,83]. Secondly, the intestines contain neutrophils that can trap and eliminate the bacteria in an oxidative burst. In addition, enterocytes and the intestinal Paneth cells secrete antimicrobial peptides, immunoglobulins, and cytokines (e.g., interleukin-33, thymic stromal lymphopoietin, or transforming growth factor-β) into the mucus layer that play an important role in ensuring tolerance to commensal bacteria and defense against pathogenic bacteria [83,84]. Finally, the dendritic and microfold cells sample bacterial antigens and induce inflammatory cascades directed against these pathogens [82,83]. The dendritic cells can also induce local hepcidin expression upon microbial stimulation, which sequesters iron, making it unavailable for bacterial growth [85].

In addition to the intestinal defensive mechanisms, commensal bacteria play an important role in maintaining a healthy intestinal barrier. Certain types of commensal bacteria, primarily Firmicutes, produce short-chain fatty acids (SCFAs) that fuel the intestinal epithelial cells ensuring effective cell regeneration and assembly of tight junction proteins. In addition, SCFAs can induce regulatory T cell activation and anti-inflammatory cytokine expression, the growth of tolerogenic dendritic cells, and modulate B cell maturation by enhancing affinity maturation of protective IgA^+^ plasma cells [82,83,86]. Despite these defensive mechanisms, some commensal opportunistic bacteria—pathobionts—have the potential to damage the epithelium. An increase in pathobionts leads to a reduction in SCFAs, degradation of the mucus layer, and invasion of the epithelial layer, which activates pro-inflammatory cascades leading to cell and tissue injury [82]. A damaged intestinal layer also increases the risk of bacterial translocation, which has even been observed in conditions characterized by low-grade inflammation, such as Chronic Fatigue Syndrome [87].

Alterations in the complex interactions between the intestinal cells and the microbiota have been implicated in various diseases, including the pathogenesis of IBD [88,89]. Previous studies have reported reduced intestinal microbial richness (⍺-diversity), lower abundances of Firmicutes and Bacteroidetes, a higher abundance of facultative anaerobes, and a predominantly inflammatory intestinal metabolome in patients with IBD [88]. Since dysbiosis is already present in patients with IBD, consensus-based guidelines advise against prescribing oral iron therapy in active IBD due to the potential for disease exacerbation, whether due to changes in the microbiota or an increase in local or systemic oxidative stress [5]. However, the guidelines do not mention the effect of iron deficiency or intravenous iron on the intestinal microbiota, and the potential interplay with oxidative stress.

### 4.1 Iron deficiency and the intestinal microbiota

Iron is essential for the host but also for the intestinal bacteria. Bacteria rely on ferrous iron transporters and siderophores, low-molecular-weight iron chelators such as enterobactin, to capture unbound dietary iron required for their survival. The siderophores are captured by receptors located on the bacteria and are then internalized [90]. Humans produce lipocalin-2 to capture enterobactin as a defense mechanism, but some bacteria can escape this defense by expressing stealth siderophores: salmochelin or aerobactin. In addition, bacteria can acquire iron by utilizing hemophores and siderophores produced by other bacteria and express receptors for host lactoferrin and transferrin, which allow the bacteria to strip the iron bound to host lactoferrin or transferrin [90,91]. Like humans, bacteria are susceptible to injury by ROS, and to prevent oxidative stress, they use iron exporters, antioxidant enzymes, and iron-storage proteins [90].

All intestinal bacteria, except for *Lactobacillus,* were thought to need iron for survival [92]. However, it seems that all commensal bacteria compete for iron with each other and the host, which is an integral part of intestinal homeostasis to stop pathobionts from colonizing the intestines. For instance, *Bifidobacteriaceae* can bind iron to its surface and act as an iron chelator to prevent ROS generation and reduce the available iron for other bacteria within the intestine [93,94]. Lipocalin-2 levels also increase during intestinal inflammation, suggesting that iron acquisition might be one of the virulence-associated mechanisms [95]. Recently, it has been shown that *Lactobacillus* bacteria can sense iron scarcity and release reuterin and 1,3-diaminopropane to downregulate signaling by HIF-2⍺, which attenuates iron absorption by the host. Inhibition of HIF-2⍺ signaling leads to lower Dcytb and DMT1 expression and increased ferritin expression, which stimulates host iron storage rather than iron absorption [96]. Interestingly, when *Lactobacillus* bacteria were suppressed following antibiotic therapy, it increased gene expression for iron uptake proteins and attenuated anemia in mice [96].

Unsurprisingly, iron deficiency and iron-deficient diets have been associated with significant alterations in the intestinal microbiota. In murine models and *in vitro* studies, iron-deficient diets have been associated with lower fecal SCFA concentrations and lower abundances of SCFA-producing bacteria, such as *Roseburia* spp*.* and *Eubacterium rectale* [91]. The reduction in SCFAs increases luminal pH, which has been associated with increased virulence of pathogenic bacteria [91]. Furthermore, the decrease in *Roseburia* spp. was also accompanied by an increase in other SCFA-producing bacteria, such as Clostridium cluster IV group, which might explain why some authors observed a decrease in intestinal butyrate levels but no change in acetate and propionate levels during periods of iron deficiency [96,97]. The depletion of butyrate-producing bacteria has also been shown to increase nitrate levels favoring the growth of *Enterobacteriaceae* [98]. These findings suggest that iron deficiency negatively affects the composition and function of the intestinal microbiota. Since bacteria need iron for survival, it is postulated that iron supplementation, like iron deficiency, might negatively affect the intestinal microbiota. In the following section, we summarize the available evidence from a literature search, described in Supplementary Table S1, with an emphasis on human studies.

### 4.2 The effect of iron therapy on the intestinal microbiota

The evidence from *in vitro* studies and murine models is inconsistent. In mice, oral iron at a dose of 98 mg/kg has been shown to induce intestinal damage and carcinogenesis [99]. It has been hypothesized that excess fecal iron induces intestinal inflammation that, in turn, generates RNS, which are more favorable for pathobionts such as *Enterobacteriaceae* [100]. In mice with dextran sodium sulfate-induced colitis, oral hemin supplementation rather than systemic hemin administration reduced ⍺-diversity, Firmicute abundance, and increased Proteobacteria [101]. Similar findings along with colitis exacerbation were observed in other murine models [[102], [103], [104]]. In contrast, other studies did not observe these effects [105]. For instance, Dostal et al. reported that iron supplementation after an iron-deficient diet restored beneficial intestinal bacteria and SCFA levels, reduced opportunistic pathogen abundance, and did not affect the severity of colitis [106,107]. Iron supplementation with or without probiotics was also associated with a decrease in *Enterobacteriaceae* [108,109]. The evidence also suggests that some iron formulations, such as oral nano or sucrosomial iron, might be less available to the microbes and will not affect the intestinal microbiota [110,111]. It is unclear whether these findings can be extrapolated to humans, given the differences in the intestinal microbiota and the relative concentration of iron supplementation between humans and animals.

The evidence from human studies is scarce, as illustrated by an overview of the studies conducted in the past 23 years (Supplementary Tables S3–5). Studies with infants and children were performed primarily in developing countries. Four studies found no significant effect of iron therapy on the intestinal microbiota diversity or composition [[112], [113], [114], [115]]. Studies by Tang et al. and Owolabi et al. showed that iron supplementation, along with other nutrients, resulted in an increased ⍺-diversity and decreased relative abundance of *Enterobacteriaceae* [116,117]. On the other hand, studies performed in toddlers with a high baseline burden of pathogenic enterobacteria found increased inflammatory parameters or facultative anaerobes, such as the genus *Escherichia/Shigella* [[118], [119], [120]]. Authors also observed reduced relative abundances of *Bifidobacterium* and *Lactobacillus*, consistent with studies showing an increase in *Lactobacillus* during iron deficiency. Overall, the evidence is inconsistent; however, iron supplementation alone or with other nutrients is associated with increased facultative anaerobes in infants and toddlers with a high baseline burden of enteropathogens.

In children, only four studies have investigated the effect of iron supplementation on the intestinal microbiota. In two studies, supplementation with ≤55 mg ferrous fumarate or sulfate did not influence the intestinal microbiota, production of SCFAs, or changes in fecal calprotectin [121,122]. A study conducted in Bangladesh also showed no effect, but *Bifidobacterium* and *Lactobacillus* were negatively correlated with iron concentration in the tube-well water [123]. In contrast, Zimmermann et al. supplemented children with cookies containing 20 mg electrolytic iron four times per week and observed the following changes: increased fecal calprotectin, a decrease in lactobacilli, and an increase in enteric pathogens, such as *Salmonella* spp. or *E. coli* [124]. Relative abundances of bifidobacteria and *Bacteroides* did not change significantly. To summarize, findings in children are inconsistent, but some observations are similar to findings from studies with infants and toddlers—a decrease in *Bifidobacterium, Lactobacillus*, and an increase in *Enterobacteriaceae*.

Furthermore, nine studies have been performed with healthy and pregnant women or patients (Supplementary Table S5). Alterations in the relative abundances rather than microbiota diversity were observed in most studies. In a study with 159 pregnant women, low-dose iron supplementation (<60 mg/day) was associated with more abundant SCFA-producing bacteria compared with women who received >60 mg/day ferrous sulfate [125]. The decrease in SCFAs was also observed in 45 otherwise healthy patients with IDA, who received 200 mg t.i.d. ferrous fumarate [126]. In patients on hemodialysis, intravenous and oral iron both affected the microbiota but in different patterns. Daily ≥200 mg doses of oral iron were associated with reduced ⍺-diversity and SCFA-producing bacteria within the Firmicutes phylum, an increase in the *Veillonella* sp. and *Ruminococcus torques* group, which have been associated with a disrupted gut barrier [127,128]. Compared with oral iron, *Lactobacillus* and *Vagococcus* spp. were less abundant after repeated iron sucrose infusions [127].

Finally, a study in patients with IBD compared oral iron with intravenous iron therapy—both iron therapies did not affect intestinal inflammation or disease activity [129]. Oral and intravenous iron therapies showed distinct changes in the intestinal microbiota but did not overrule the IBD-specific pattern observed by the authors, which included a decrease in the relative abundance of Clostridiales spp. When comparing the effects of 300 mg b d. ferrous sulfate versus 300 mg intravenous iron sucrose, *Bifidobacterium* was increased after oral iron therapy but other SCFA-producing bacteria—i.e., *Faecalibacterium prausnitzii, Ruminococcus bromii, Dorea* sp*.*—were decreased [129]. Similar results were observed by Mahalhal et al. when comparing iron supplementation with 200 mg b d. ferrous fumarate and 30 mg b.d. ferric maltol. In contrast, oral ferric maltol did not affect the intestinal microbiota [130].

In conclusion, the evidence on the effect of iron therapy on the intestinal microbiota is scarce and inconsistent, especially in patients with IBD. Like studies examining iron therapy and redox status, these studies also used various iron formulations, dosages, supplementation regimens, and methods to profile the intestinal microbiota. Consequently, it is difficult to generalize and interpret these findings due to the limitations of the published literature. Nevertheless, oral and intravenous iron therapy affects the intestinal microbiota, yet the clinical significance or duration of these alterations has not been established.

### 4.3 The interplay of the intestinal microbiota and redox status during iron therapy

The interaction between iron, the intestinal microbiota, and redox status is not yet completely understood. Nonetheless, disturbances in oxygen tension near the colonic epithelium and the mucous layer in patients with IBD might contribute to iron-related ROS generation [91,131]. In addition, inflammatory responses stemming from intestinal infection or dysbiosis lead to a release of reactive species from infiltrated neutrophils [91]. This effect is further amplified by the presence of luminal iron, whether from oral iron supplements, nutritional iron, or the presence of blood (e.g., from ulcerating mucosa). The inflammatory environment can initiate a vicious cycle where ROS catalyze the formation of nitrates, S-oxides, and N-oxides, serving as alternative respiration sources for luminal pathogens like *S. typhimurium*, thereby exacerbating intestinal dysbiosis and oxidative stress [91,132]. Unlike pathogenic bacteria, obligate anaerobes usually lack oxidoreductases to utilize these alternative respiration sources [91,132]. Instead, these bacteria, such as *F. prausnitzii*, may leverage more sophisticated forms of anaerobic metabolism that can use antioxidant compounds (e.g., thiols) for extracellular electron transfer through shuttling electrons to oxygen [133,134]. In this way, obligate anaerobes tend to reduce their oxygenated microenvironments and could prevent local oxidative stress, thereby stimulating their growth at the oxic-anoxic interphase of the human gut.

Moreover, inflammation-induced hepcidin expression causes iron restriction within enterocytes that can act as an iron source for invading pathogens, promoting inflammatory responses, intestinal dysbiosis, oxidative stress, and cellular or tissue damage. In addition, excess iron can induce ferroptosis—a type of iron-dependent programmed cell death—characterized by iron overload, morphological changes, accumulation of reactive species, and lipid peroxidation [135]. We speculate that increased cellular iron stores, whether from oral or intravenous iron supplementation or hepcidin-induced iron restriction, can render cells more susceptible to ferroptosis and promote the vicious cycle described in the previous section. Patients with IBD also often suffer from folate deficiency that influences cellular resistance to ferroptosis, given that GCH1-mediated BH4 biosynthesis is vital for ferroptosis resistance through remodeling lipidomic composition and suppressing lipid peroxidation [135]. Finally, ferroptosis-associated genes, such as acyl-CoA synthetase family member 2, GPX4, LPCAT3, NCOA4, and glucose-6-phosphate dehydrogenase, have been shown to be dysregulated in IBD [136]. This dysregulation might make patients more susceptible to ferroptosis and contribute to the intestinal dysbiosis and oxidative stress. In conclusion, the damaged intestinal epithelial layer in IBD creates a favorable microenvironment for iron toxicity and its consequences. However, it remains unclear how intravenous iron might influence these processes and whether excess luminal iron can lead to systemic oxidative stress.

## 5 Clinical implications for inflammatory bowel disease

Despite the limitations of the existing literature, we can conclude that oral and intravenous iron supplementation alters the intestinal microbiota. Intravenous iron therapy is associated with increased oxidative stress, especially compared to oral iron therapy. While iron excess is harmful, the significance of appropriate iron therapy and its effects on the redox status and the microbiota in terms of their duration and clinical impact must be investigated further.

Moreover, the alterations in the intestinal microbiota should not immediately be characterized as pathological and might even serve as potential therapeutic targets. To illustrate, *Lactobacillus plantarum* has been shown to promote iron absorption in women with IDA, and SCFAs have been shown to reduce the intestinal pH to improve intestinal iron bioavailability, as described in a recent review [137,138]. Modulating intestinal iron absorption may be a promising strategy to treat iron deficiency and iron overload, as evidenced by *Lactobacillus*-produced reuterin and 1,3-diaminopropane, which attenuated iron absorption by the host, and the positive effect on anemia after antibiotic treatment that decreased *Lactobacillus* and reuterin [96].

However, until more evidence is available, it is important to remember that not only iron excess but also iron deficiency affects redox status and the intestinal microbiota, as summarized in Fig. 4. Therefore, patients with IBD must be treated for NAID and IDA with oral or intravenous iron. It has previously been suggested that patients with IBD should not be treated with oral iron due to the possibility of hepcidin-mediated iron malabsorption; however, studies have shown that even in inflammatory states, iron status is the primary determinant of hepcidin levels [[139], [140], [141], [142]]. Oral iron should still be considered an effective therapy in patients with IBD. However, whether oral or intravenous iron is safer regarding the effect on redox status and the intestinal microbiota remains to be discovered.

In conclusion, iron deficiency negatively impacts clinical outcomes, QoL, redox status, and intestinal microbiota. Appropriate therapy might mitigate some or all of these effects. Current data are scarce, inconsistent, and do not provide a definitive answer. Nevertheless, intravenous and oral iron therapy influences the redox status and the intestinal microbiota. It is imperative to discern IDA from other types of anemia before prescribing iron therapy to prevent untargeted or excessive iron supplementation and potential side effects. Patients with IBD are more susceptible to common infections, including gastrointestinal infections, than the general population [143]. Gastrointestinal infections—most frequently caused by *C. difficile*, different *E. coli* types, or viruses—are also common causes of IBD relapses or steroid-refractory IBD in approximately 30% of symptomatic patients; therefore, ruling out infections before prescribing iron is essential, given the evidence relating to enteropathogenic expansion with iron therapy [144]. Low-dose and intermittent iron supplementation is recommended in patients with quiescent IBD, preferably with non-ferrous salt compounds due to their lower potential for inducing oxidative stress and alterations in the intestinal microbiota. However, whether oral or intravenous iron should be prescribed in active IBD remains debatable, as both modalities are effective but have different effects on redox status and intestinal microbiota. Further research is needed to investigate the significance of these effects in terms of their duration and clinical relevance.

## 6 Future directions

The discovery of hepcidin approximately 20 years ago has shed light on iron metabolism and provided evidence for improving current therapies, such as intermittent oral iron supplementation and prioritizing anti-inflammatory therapy in patients with functional iron deficiency or anemia of chronic disease [[26], [27], [28],^79^]. In patients with IBD, the coexistence of inflammation, hypoxia, and iron deficiency complicates NAID and IDA diagnosis and therapy. Further research is needed to optimize and personalize iron therapy. Table 1 presents an overview of areas necessitating further investigation, including diagnostic markers, optimization of enteral iron uptake, predicting the response to iron therapy, the most effective iron modality or supplementation regimen, and the prevention of secondary injury mechanisms such as oxidative stress.

**Table 1:** Areas necessitating further investigation to improve the diagnostic and therapeutic processes for iron deficiency in patients with Inflammatory Bowel Disease.

Area		Rationale
Iron metabolism	Hepcidin regulation Ferroportin regulation Heme iron uptake Intracellular iron regulation Iron absorption regulation by host-microbiota interactions	Much is still unknown about iron metabolism, including the factors that regulate key proteins and their expression, function, or even the role of other organs, e.g., the pancreas. Establishing regulatory pathways will help understand the relationship between iron status and disease.

Physiology	ID classification by different stages and severity Effect of iron status on different physiological functions, e.g., immune system Association between iron status, hypoxia, and oxidative stress Association between iron status and the intestinal microbiota Association between iron status and other deficiencies, e.g., zinc	There are no definitions of mild, moderate, or severe ID. Establishing different phases of ID and its relation to various physiological processes will help to improve the diagnostic and therapeutic processes. In addition, the effect of hypoxia on iron deficiency and oxidative stress should be explored further, given their frequent co-existence in patients with IBD.

Diagnosis	Assessing systemic iron status Diagnostic biomarker standardization Assessing abnormalities in iron metabolism	Currently used biomarkers are susceptible to inflammation, which impacts the diagnostic accuracy and the utility of a common cut-off point. The discovery and validation of new diagnostic biomarkers and their quantification methods will help identify patients needing treatment in an accurate and timely manner. Currently, assessment of congenital abnormalities regarding iron status is limited; understanding and diagnosing abnormallities in iron metabolism will aid prescribing the appropriate therapy.

Therapy	Appropriate nutrition Optimizing iron therapy Predicting response to iron therapy	More research is needed to establish guidelines for appropriate nutrition to optimize iron status in patients with co-morbidities, a history of abdominal surgery, use of medications (e.g., PPIs), and active IBD. Improving oral and intravenous iron therapy is necessary to ensure therapeutic compliance, ID recurrence prevention, and optimization of clinical outcomes.

ID: iron deficiency, IBD: Inflammatory Bowel Disease, PPI: proton-pump inhibitor.

Much is still unknown about the regulation of hepcidin expression, the genes involved in iron absorption and homeostasis, changes in host and the intestinal microbiota in times of iron deficiency and iron supplementation, and the interplay between oxidative stress and the intestinal microbiota. Adequately powered prospective studies are essential to better understand iron metabolism and improve care for patients with IBD and abnormal iron status.

## Funding

This review was not funded.

## Authors’ contributions

RL performed the literature search and appraised it. RL summarized the findings, made the figures, and wrote the first draft of the manuscript. All authors contributed to the manuscript revision, read and approved the final manuscript.

## Declaration of competing interest

Outside of the submitted work, GD has received research grants from Royal DSM, Janssen Research and Development LLC, and received speaker's fees from Janssen Pharmaceuticals, Takeda, Pfizer, and AbbVie. Outside of the submitted work, AEvdMdJ has received unrestricted research grants from Galapagos, Norgine Ltd., Vedanta, and Nestle; and speaker fees from Galapagos, Tramedico, Takeda, Ferring, and Janssen Pharmaceuticals. Outside of the submitted work, RL has received travel expenses from Galapagos, served on the advisory board and received speaker's fees from Cablon Medical. ARB has received a research grant from Janssen Research and Development LLC and received speaker's fees from AbbVie, outside the submitted work. All other authors have no conflicts of interest to declare.

## Data Availability

No data was used for the research described in the article.
