# Supplementary material for: The effect of iron therapy on oxidative stress and intestinal microbiota in inflammatory bowel diseases: A review on the conundrum
Source: Redox Biol. 2023 Oct 30;68:102950. doi: 10.1016/j.redox.2023.102950 (PMC10643537; doi:10.1016/j.redox.2023.102950)
Supplement: Multimedia component 1 [file mmc1.docx]

**The effect of iron therapy on oxidative stress and intestinal microbiota in inflammatory bowel diseases:**

**a review on the conundrum**

R. Loveikyte^1,2^, A.R. Bourgonje^2,3^, H. van Goor^4^, G. Dijkstra^2,#^, A.E. van der Meulen – de Jong^1,#^

Affiliations:

^1^ Department of Gastroenterology and Hepatology, Leiden University Medical Center, Leiden, the Netherlands

^2^ Department of Gastroenterology and Hepatology, University Medical Center Groningen, University of Groningen, Groningen the Netherlands

^3^ The Henry D. Janowitz Division of Gastroenterology, Department of Medicine, Icahn School of Medicine at Mount Sinai, New York, NY, United States

^4^ Department of Pathology and Medical Biology, University Medical Center Groningen, University of Groningen, Groningen the Netherlands

^#^ shared last authors

**SUPPLEMENTARY TABLES**

**Supplementary table S1: search strategy for the literature search**

| **Database** | **Period** | **Search strategy** |
| --- | --- | --- |
| Pubmed | 2000 – 2023 (April) | “((("Gastrointestinal Microbiome"[Mesh] OR "Gastrointestinal Microbiome"[tw] OR "Gastrointestinal Microbiomes"[tw] OR "Enteric Bacteria"[tw] OR "Gastric Microbiome"[tw] OR "Gastric Microbiomes"[tw] OR "Gastrointestinal Flora"[tw] OR "Gastrointestinal Microbial Communities"[tw] OR "Gastrointestinal Microbial Community"[tw] OR "Gastrointestinal Microbiomes"[tw] OR "Gastrointestinal Microbiota"[tw] OR "Gastrointestinal Microbiotas"[tw] OR "Gastrointestinal Microflora"[tw] OR "Gut Flora"[tw] OR "Gut Microbiome"[tw] OR "Gut Microbiomes"[tw] OR "Gut Microbiota"[tw] OR "Gut Microbiotas"[tw] OR "Gut Microflora"[tw] OR "Intestinal Flora"[tw] OR "Intestinal Microbiome"[tw] OR "Intestinal Microbiomes"[tw] OR "Intestinal Microbiota"[tw] OR "Intestinal Microbiotas"[tw] OR "Intestinal Microflora"[tw] OR "Microbiota"[Mesh] OR "Microbiota"[tw] OR "Microbiotas"[tw] OR "Microbial Community"[tw] OR "Microbial Communities"[tw] OR "Microbiome"[tw] OR "Microbiomes"[tw] OR "Microbial Consortia"[tw] OR "Mycobiome"[tw] OR "Periphyton"[tw] OR "Virome"[tw] OR "Oxidative Stress"[Mesh] OR "Oxidative Stress"[tw] OR "Oxidative Stress*"[tw] OR "Antioxidative Stress"[tw] OR "Antioxidative Stress*"[tw] OR "Nitrosative Stress"[tw] OR "Nitrosative Stress"[tw] OR "Oxidative Cleavage"[tw] OR "Oxidative Damag*"[tw] OR "Oxidative Damage"[tw] OR "Oxidative DNA Damage"[tw] OR "Oxidative DNA Damages"[tw] OR "Oxidative Injuries"[tw] OR "Oxidative Injury"[tw] OR "Oxidative Stress*"[tw] OR "Protein Carbamylation"[tw] OR "Protein Carbonylation"[tw]) AND ((("Iron"[majr] OR "Iron Compounds"[majr] OR "iron"[ti] OR "ferric"[ti]) AND ("Administration, Oral"[majr] OR "oral"[ti] OR "orally"[ti] OR "buccal"[ti] OR "sublingual"[ti] OR "Administration, Intravenous"[majr] OR "Intravenous"[ti] OR "Intravenous*"[ti])) OR "oral iron"[ti] OR "oral ferric"[ti] OR "intravenous iron"[ti] OR "intravenous ferric"[ti])) OR (("Gastrointestinal Microbiome"[majr] OR "Gastrointestinal Microbiome"[ti] OR "Gastrointestinal Microbiomes"[ti] OR "Enteric Bacteria"[ti] OR "Gastric Microbiome"[ti] OR "Gastric Microbiomes"[ti] OR "Gastrointestinal Flora"[ti] OR "Gastrointestinal Microbial Communities"[ti] OR "Gastrointestinal Microbial Community"[ti] OR "Gastrointestinal Microbiomes"[ti] OR "Gastrointestinal Microbiota"[ti] OR "Gastrointestinal Microbiotas"[ti] OR "Gastrointestinal Microflora"[ti] OR "Gut Flora"[ti] OR "Gut Microbiome"[ti] OR "Gut Microbiomes"[ti] OR "Gut Microbiota"[ti] OR "Gut Microbiotas"[ti] OR "Gut Microflora"[ti] OR "Intestinal Flora"[ti] OR "Intestinal Microbiome"[ti] OR "Intestinal Microbiomes"[ti] OR "Intestinal Microbiota"[ti] OR "Intestinal Microbiotas"[ti] OR "Intestinal Microflora"[ti] OR "Microbiota"[majr] OR "Microbiota"[ti] OR "Microbiotas"[ti] OR "Microbial Community"[ti] OR "Microbial Communities"[ti] OR "Microbiome"[ti] OR "Microbiomes"[ti] OR "Microbial Consortia"[ti] OR "Mycobiome"[ti] OR "Periphyton"[ti] OR "Virome"[ti] OR "Oxidative Stress"[majr] OR "Oxidative Stress"[ti] OR "Oxidative Stress*"[ti] OR "Antioxidative Stress"[ti] OR "Antioxidative Stress*"[ti] OR "Nitrosative Stress"[ti] OR "Nitrosative Stress"[ti] OR "Oxidative Cleavage"[ti] OR "Oxidative Damag*"[ti] OR "Oxidative Damage"[ti] OR "Oxidative DNA Damage"[ti] OR "Oxidative DNA Damages"[ti] OR "Oxidative Injuries"[ti] OR "Oxidative Injury"[ti] OR "Oxidative Stress*"[ti] OR "Protein Carbamylation"[ti] OR "Protein Carbonylation"[ti]) AND ((("Iron"[mesh] OR "Iron Compounds"[Mesh] OR "iron"[tw] OR "ferric"[tw]) AND ("Administration, Oral"[Mesh] OR "oral"[ti] OR "orally"[ti] OR "buccal"[ti] OR "sublingual"[ti] OR "Administration, Intravenous"[Mesh] OR "Intravenous"[ti] OR "Intravenous*"[ti])) OR "oral iron"[tw] OR "oral ferric"[tw] OR "intravenous iron"[tw] OR "intravenous ferric"[tw])))”  In addition, other relevant articles have been found by the snowball method: using one article to find other older or more recent literature on the same subject. |

**Supplementary table S2: High-level summary of findings regarding the effect of iron supplementation on redox status in adults.**

| **Author** | **Population** | **Intervention** | **The effect of intervention on redox status** |
| --- | --- | --- | --- |
| **Healthy volunteers** | | | |
| Rooyakkers et al., 2002^1^ | Healthy male volunteers  *(n* = 20) | 100 mg i.v. ferric saccharate | Infusion of ferric saccharate induced a >4-fold increase in NTBI.  The generation of superoxide in whole blood increased significantly 10 and 240 min after infusion of ferric saccharate by 70% and 53%, respectively. |
| Schümann et al.,  2005^2^ | Healthy volunteers  (*n* = 3) | 120 mg ferrous sulfate for 7 days | Iron supplementation: ↔︎ SOD, hemoxygenase-1, catalase, TBARS, carbonyl proteins, TOC  Only urinary TBARS, 8-OHdG, and F_2_-isoprostanes showed transient increases. |
| Colpo et al.,  2008^3^ | Healthy men  (*n* = 9) | Men were supplemented with single doses: 1. 2 g ascorbic acid 2. 150 mg iron carbonyl 3. 2 g ascorbic acid and 150 mg iron carbonyl  Washout period – 15 days | Ascorbic acid supplementation: ↓ Erythrocyte MDA ↔︎ NPSH, DCHF oxidation, blood δ -ALA-D, plasma GPx activities and erythrocyte SOD  Ascorbic acid and iron supplementation: ↑ Catalase activity from erythrocytes  ↓ Erythrocyte MDA |
| Orozco et al.,  2012^4^ | Healthy men  (*n* = 10) | For 6 days each, separated by 10-day washout: 1. Daily 100 mg elemental iron as ferrous sulfate 2. Daily 100 mg elemental iron as NaFeEDTA 3. Daily 100 mg elemental iron as iron polymaltose | Regardless of iron formulation, iron supplementation: ↑ Residual non-heme iron in stool ↑ Fecal ROS |
| Orozco et al.,  2012^5^ | Healthy men  (*n* = 17) | For 7 days each, separated by 12-day washout: 1. Daily 120 mg ferrous sulfate 2. Daily 120 mg ferrous sulfate in 5 mL refined palm oil 3. Daily 120 mg ferrous sulfate in 5 mL palm oil combined with CTCMC | Iron supplementation: ↔︎ Urinary TBARS and 8-OHdG |
| Wang et al.,  2017^6^ | Healthy volunteers  (*n* = 21) | Randomized to receive one dose: 1. 25 mg iron-denatured whey protein microspheres  2. 25 mg ferrous sulfate Washout period – 28 days | Iron-denatured whey protein showed better absorption than ferrous sulfate; it also caused less ROS generation and better gut epithelial (HT29) cell viability than equimolar ferrous sulfate. |
| Abiri et al.,  2021^7^ | Women with vitamin D deficiency (*n* = 87) | For 12 weeks, randomized to receive: 1. 1000 IU/day cholecalciferol + placebo 2. Cholecalciferol + 27 mg/day ferrous fumarate | Iron co-supplementation with cholecalciferol: ↔︎ TAC, MDA |
| **Patients** | | | |
| Erichsen et al.,  2003^8^ | Patients with active CD (*n* = 10)  Healthy volunteers (*n* = 10) | 120mg ferrous fumarate for 7 days | Compared with healthy volunteers, iron therapy in CD:  ↓ cysteine, GSH |
| Erichsen et al.,  2005^9^ | Patients with IBD (*n* = 41) | For 14 days, randomized to receive: 1. 100 mg b.i.d. ferrous sulfate  2. 200 mg o.d. iron hydroxide polymaltose complex | Ferrous sulfate supplementation: ↑ MDA ↔︎ Urine 8-iso-PGF2α and plasma antioxidants (i.e., β-carotene, GSH, cysteine, cysteinyl-glycine, homocysteine, vitamins A, C, and E) Iron hydroxide polymaltose complex supplementation: ↔︎ MDA, urine 8-iso-PGF2α and plasma antioxidants |
| Erichsen et al.,  2005^10^ | Patients with IBD (*n* = 19) | 1. 120 mg o.d. ferrous fumarate 2. 200 mg i.v. iron sucrose thrice of a period of 14 days  Washout period – 6 weeks | Ferrous fumarate: ↔︎ MDA, β-carotene, GSH, cysteine, cysteinyl-glycine, vitamins A, C, and E Iron sucrose: ↑ MDA  ↓ β-carotene, vitamin C |
| Lasocki et al.,  2016^11^ | Critically ill patients (*n* = 38)  Healthy volunteers (*n* = 39) | 100 mg i.v. ferric hydroxide sucrose | Iron supplementation in both groups: ↑ 8-ISO, AOPP, NTBI, and MPO  Compared with critically ill, iron supplementation: ↓ GSH:GSSG ratio in healthy volunteers |
| Pérez-Peiró et al., 2021^12^ | Patients with COPD (*n* = 66) | 1. 500–1000 mg i.v. ferric carboxymaltose, dose adjusted for weight 2. Placebo | Iron supplementation: ↑ GSH, reactive carbonyls  ↓ serum MDA-protein adducts, 3-nitrotyrosine, TEAC  ↔︎ Catalase, SOD |
| Isler et al.,  2002^13^ | Patients with IDA (*n* = 28)  Healthy volunteers (*n* = 18) | 1. 567.7 mg b.i.d. ferroglycine sulphate for four months 2. 200 mg i.m. iron polymaltose every other day for a month 3. i.m. iron + daily 300 mg vitamin E for a month | SOD activity was lower in patients with anemia than healthy volunteers.  Iron supplementation: ↑ SOD (especially with oral iron) ↓ GPx (only with i.v. or i.v. + vitamin E supplementation, not with oral iron) |
| Kurtoglu et al.,  2003^14^ | Patients with IDA (*n* = 189)  Healthy volunteers (*n* = 30) | Subjects were analyzed in four groups: 1. Age- and sex-matched healthy volunteers 2. Patients with IDA not receiving any treatment at the beginning of the study 3. Patients with IDA at the sixth week of iron supplementation 4. Patients with IDA at the end of iron supplementation | Patients with IDA had higher MDA and lower catalase, SOD, and GPx levels than healthy volunteers.  Iron supplementation: ↓ MDA  ↑ Catalase, SOD, GPx  These changes were observed at the end and after six weeks of supplementation. |
| Binkoski et al.,  2004^15^ | Healthy women with low ferritin (*n* = 26) | 1. 160 mg b.d. ferrous sulfate (100 mg total elemental iron) 2. Placebo  Iron or placebo supplementation were combined with AAD (36% fat, 15% SFA) or Step 2 diet (26% fat, 7% SFA). Washout period: 2 weeks. | Iron supplementation did not affect measures of LDL oxidation.  Lag time was shorter after the women consumed the AAD diet than after the Step 2 diet. The diets did not affect the rate of oxidation or total dienes. |
| Ruivard et al.,  2006^16^ | Women with ID(A)  (*n* = 24) | For 3 months, women received: 1. 178 mg ferrous chloride daily (50 mg elemental iron) + 100 mg vitamin C 2. 178 mg ferrous chloride (50 mg elemental iron) twice weekly + 100 mg vitamin C | Daily and intermittent iron supplementation:  ↔︎ FRAP and TBARS |
| Sundaram et al.,  2007^17^ | Patients with anemia (*n* = 20)  Healthy volunteers (*n* = 16) | Patients with anemia received 200 mg t.i.d. ferrous sulfate for 1 month | MDA was higher in patients with anemia than healthy volunteers.  Iron supplementation: ↓ MDA |
| King et al.,  2008^18^ | Healthy women with ID (*n* = 12) | 98 mg iron as ferrous fumarate daily for 8 weeks | Iron supplementation: ↑ Breath ethane exhalation rates  ↑ MDA |
| Tiwari et al.,  2011^19^ | Women with IDA  (*n* = 117) Healthy women volunteers  (*n* = 60) | For 100 days, women receive: 1. Daily 100 mg ferrous fumarate and 500 µg folic acid 2. Placebo for healthy volunteers | Iron supplementation:  ↑ Catalase, SOD, GSH-Rd, TAC  ↑ LPO, protein carbonyl, conjugated dienes, LOOH, GSSH  ↓ GPx, vitamins A, C and E |
| Akarsu et al.,  2013^20^ | Patients with IDA (*n* = 60) Healthy volunteers (*n* = 20) | 1. Ferrous sulfate, 4–6 mg/kg for 3 months 2. I.m. ferric hydroxy polymaltose, given on alternate days at an average dose of 5.9 mg/kg (3–15 days of therapy) and then maintained daily 3.2–24.6 mg/kg per dose. 3. I.v. iron sucrose, dose given based on patient’s hemoglobin and weight 4. Control group (healthy volunteers without anemia) | TAC in the oral iron group was closest to the control group level.  I.v. iron reduced TAC the most compared with oral and i.m. iron supplementation. |
| Khoshfetrat et al., 2013^21^ | Women with ID (*n* = 60) | For 12 weeks, randomized to receive: 1. 50 mg/day elemental iron 2. 50 mg/day elemental iron + 500 mg/day ascorbic acid | Iron supplementation with or without vitamin C: ↑ TAC, vitamin C ↓ MDA |
| **Pregnant or lactating women** | | | |
| Lachili et al.,  2001^22^ | Pregnant women  (*n* = 54) | 1. 100 mg ferrous fumarate and 500 mg ascorbic acid once daily during the third trimester of pregnancy 2. Control group | Iron and vitamin C supplementation: ↑ TBARS  ↔︎ Antioxidant micronutrients (Zn, Se, retinol, vitamin E, and β-carotene) ↔︎ Antioxidant metalloenzymes (Cu-Zn SOD, Se-GPx) |
| Rehema et al.,  2004^23^ | Pregnant women (*n* = 19) | 1. Daily 36 mg ferrous iron 2. No iron supplementation | Iron supplementation did not affect oxidative stress markers, except for increased GSSH, compared with women not receiving iron. |
| Ma et al.,  2010^24^ | Pregnant women  (*n* = 164) | For 2 months, women were randomized to receive: 1. Placebo control group 2. Daily 60 mg iron as ferrous sulfate  3. Daily iron and 400 µg folic acid  4. Daily iron, folic acid, and 2 mg retinol + 1mg riboflavin | With exception of the placebo group, all groups: ↑ GPx activity, retinol, erythrocyte membrane fluidity ↓ MDA ↔︎ SOD |
| Han et al.,  2011^25^ | Pregnant women with IDA  (*n* = 153) | For 2 months, women were randomized to receive either:  1. Placebo control group  2. Daily 60 mg iron as ferrous sulfate  3. Daily 60 mg iron as NaFeEDTA | In both iron supplementation groups: ↑ GPx activity (especially with NaFeEDTA supplementation)  ↓ MDA ↔︎ SOD |
| Viteri et al.,  2012^26^ | Pregnant women without anemia (*n* = 100) | Women were randomized to receive: 1. Daily-weekly group: daily tablet containing 60 mg of iron, 0.2 mg of folic acid, and 1 µg of vitamin B12, followed by weekly supplementation with two tablets. Each period for 8 weeks. 2. Weekly-daily group: inverted supplementation schedule. | Daily iron supplementation: ↑ TBARS  Weekly iron supplementation reduced increases in TBARS level from daily iron supplementation. |
| Korkmaz et al.,  2014^27^ | Pregnant women  (*n* = 108) | Patients were randomized to receive: 1. Placebo group 2. Daily 400 µg folic acid 3. Daily 60 mg iron | Iron supplementation: ↑ g-Glutamyl transferase |
| Lymperaki et al.,  2015^28^ | Pregnant women  (*n* = 50) Healthy non-pregnant women (*n* = 25) | Pregnant women received supplementation with: 1. Iron 2. Folic acid 3. Iron and folic acid 4. No supplementation | Iron supplementation with or without folic acid: ↑ TAC (especially in patients co-supplemented with folic acid) |
| Jorgensen et al.,  2017^29^ | Lactating women  (*n* = 114) | For 3.5 months, women were randomized to receive: 1. Daily iron-free prenatal vitamins + placebo 2. Daily iron-free prenatal vitamins + 27 mg iron sulfate (consumed with meals) 3. Daily iron-free prenatal vitamins + 27 mg iron sulfate (consumed between meals) | No differences in oxidative stress between study groups. |
| Jacob et al.,  2020^30^ | Pregnant women with anemia (*n* = 45) | I.v. iron sucrose administered as 300 mg doses; the total dose was based on the Ganzoni formula. | Iron supplementation: ↓ SOD  ↔︎ MDA, FRAP |
| **Patients with CKD** | | | |
| Agarwal et al.,  2004^31^ | Patients with stage 3–4 CKD  (*n* = 20) | 1. 600mg b.d. N-acetylcysteine for 1 week followed by 100 mg i.v. iron sucrose. 2. Placebo for 1 week followed by 100 mg i.v. iron sucrose | I.v. iron increased plasma and urine MDA, but it returned to baseline levels within 24 hours.  NAC reduced acute generation of systemic oxidative stress. |
| Leehey et al.,  2005^32^ | Patients with CKD (*n* = 8) | 1. 125mg i.v. iron gluconate over 1h + placebo the day prior to i.v. iron and the day of i.v. iron 2. 125mg i.v. iron gluconate over 1 h + 600 mg b.d. NAC the day prior to i.v. iron and the day of i.v. iron 3. 250 mg i.v. iron gluconate over 2 h + placebo  4. 250 mg i.v. iron gluconate over 2 h + NAC  Washout period — 1 week | I.v. iron gluconate: ↑ MDA ↑ Urine MDA (only at the higher iron dose)  NAC pre-treatment had no effect on MDA levels. |
| Sezer et al.,  2007^33^ | Patients with ESRD (*n* = 68) | 100 mg i.v. ferric sucrose administered over 30 min | Iron supplementation: ↑ Serum IsoPG-F2 alpha (especially in patients with hypoalbuminemia) |
| Ganguli et al.,  2009^34^ | Patients with stage 3–4 CKD  (*n* = 59) | Patients randomized to receive: 1. 100 mg i.v. iron sucrose 2. 100 mg i.v. iron dextran 3. 125 mg i.v. sodium ferrifluconate complex in sucrose (SFGC) | I.v. iron supplementation: ↑ MDA (especially after SFGC administration) |
| Kassianides et al., 2021^35^ | Patients with CKD stage 3–5  (*n* = 40) | Randomized to receive single infusion: 1. 200 mg i.v. iron dextran 2. 200 mg i.v. iron sucrose 3. 200 mg i.v. ferric derisomaltose 4. 1000 mg i.v. ferric derisomaltose | All i.v. iron infusions increased TBARS within hours of the infusion; however, the levels of TBARS and NTBI normalized within 1 week. |
| Kassianides et al., 2022^36^ | Patients with CKD stage 3–5  (*n* = 54) | Randomized to receive a single dose: 1. Placebo 2. 1000 mg i.v. ferric derisomaltose | Ferric derisomaltose: ↓ F_2_-isoprostane  ↔︎ TBARS |
| Kassianides et al., 2022^37^ | Patients with CKD stage 3–5  (*n* = 36) | Pooled analysis of two trials. Patients received a single 1000 mg dose of i.v. ferric derisomaltose | Ferric derisomaltose: ↔︎ TBARS |
| **Patients on maintenance dialysis** | | | |
| Roob et al.,  2000^38^ | Patients on hemodialysis  (*n* = 22) | 1. 100 mg of i.v. ferric hydroxide sucrose complex during hemodialysis  2. 100 mg of i.v. ferric hydroxide sucrose complex during hemodialysis + a single oral dose of 1200 IU of all-rac-⍺-tocopheryl acetate taken 6 h before the hemodialysis session Washout period — 7 days | Iron supplementation: ↑ BDI, MDA, MDA: cholesterol ratio, peroxidases:cholesterol ratio Vitamin E co-supplementation partially attenuated these increases. |
| Herrera et al.,  2001^39^ | Patients on hemodialysis  (*n* = 9) | Patient received the following treatments in a random order: 1. 100 mg i.v. iron saccharate over 1 hour + 0.3 mg/kg Fe melatonin 1 hour before i.v. iron administration 2. 100 mg i.v. iron saccharate over 1 hour + placebo 1 hour before i.v. iron administration  3. 4000 units i.v. rHuEPO + melatonin 4. 4000 units i.v. rHuEPO + placebo | I.v. iron supplementation and rHuEPO resulted in transient: ↑ MDA ↓ GSH, catalase activity  Co-administration of melatonin prevented these changes. |
| Salahudeen et al.,  2001^40^ | Patients on hemodialysis  (*n* = 22) | 700 mg bolus of i.v. iron dextran on a non-hemodialysis day | ↑Esterified F_2_-isoprostanes in plasma lipoproteins  ↔︎ Plasma free F_2_-isoprostanes |
| Tovbin et al.,  2002^41^ | Patients on hemodialysis  (*n* = 19) | 100 mg i.v. iron saccharate administered over 1 hour after 3.5 hours of high-flux dialysis | Iron supplementation: ↑ AOPP level  ↔︎ TAC, thiols, di-tyrosine |
| Cavdar et al.,  2003^42^ | Patients on hemodialysis  (*n* = 13) | During three hemodialysis sessions, patients received: 1. Hemodialysis without iron administration (control) 2. 20 mg i.v. iron hydroxide sucrose at the end of dialysis 3. 100 mg i.v. iron hydroxide sucrose at the end of dialysis | Iron administration did not cause additional oxidant stress than hemodialysis alone. |
| Michelis et al.,  2003^43^ | Patients on hemodialysis  (*n* = 17) | 1. No iron during hemodialysis (control) 2. 62.5mg i.v. iron gluconate  3. 125 mg i.v. iron gluconate | ↑ Carbonylated fibrinogen with one dose of 125 mg i.v. iron gluconate  ↔︎ Carbonylated fibrinogen with one dose of 62.5 mg i.v. iron gluconate |
| Anraku et al.,  2004^44^ | Patients on hemodialysis  (*n* = 22) | 1. 40 mg i.v. saccharated ferric oxide after every dialysis session for four weeks 2. Control group treated with saline | I.v. iron: ↑ Disulfide and oxidized form of albumin  ↑ Plasma protein carbonyls |
| Tiranathanagul  et al., 2004^45^ | Patients on hemodialysis  (*n* = 19) | 100 mg i.v. iron sucrose every 2 weeks either as a rapid or a slow infusion | Iron supplementation as rapid or slow infusion: ↔︎ TBARS, TAC, thiols, vitamin E |
| Driss et al.,  2005^46^ | Patients on hemodialysis  (*n* = 65) | Study A: 1. Received weekly 100 mg i.v. iron polymaltose 2. No iron therapy for more than two months  Study B: 1. One hemodialysis session without iron supplementation 2. One hemodialysis session with 100 mg i.v. iron polymaltose administered over 4 hours | In study A, NTBI was detected in 41% of the patients and the proportion of NTBI-positive patients was the same whether or not they received iron therapy.  In study B, iron supplementation: ↔︎ TAC, 8-OHdG, hydroperoxidases |
| Hodkova et al.,  2005^47^ | Patients on hemodialysis  (*n* = 20) | One hemodialysis session without iron supplementation followed by another hemodialysis session with 62.5 mg i.v. sodium ferric gluconate | Iron supplementation: ↑ PAPP-A |
| Mimić-Oka et al., 2005^48^ | Patients on hemodialysis  (*n* = 19)  Healthy volunteers  (*n* = 20) | 625 mg i.v. ferrous gluconate over the course of 10 hemodialysis sessions | Iron supplementation: ↑ MDA, CRD ↓ Non-protein SH thiol groups  ↔︎ SOD and GPx activities |
| Eiselt et al.,  2006^49^ | Patients on hemodialysis  (*n* = 20) | Patients with vitamin C deficiency were treated with 100 mg i.v. iron sucrose during dialysis.  After vitamin C repletion, patients received either: 1. 100mg i.v. iron sucrose 2. 100mg i.v. iron sucrose and continuous 2 mg/min (total dose 480 mg) i.v. vitamin C throughout dialysis 3. Saline 4. Saline and continuous 2 mg/min i.v. vitamin C | Iron supplementation in patients with or without vitamin C deficiency: ↑ TBARS ↔︎ GSH  Vitamin C supplementation in addition to i.v. iron supplementation resulted in a greater increase in TBARS. |
| De Vecchi et al.,  2007^50^ | Patients on peritoneal dialysis  (*n* = 20) | 1. 62.5 mg i.v. iron gluconate, administered in 1–2min 2. 62.5 mg i.v. iron gluconate, administered in 30 min  Washout period: 15–60 days | I.v. iron gluconate: ↔︎ ROS and TAC (no differences between slow and fast administration of i.v. iron) |
| Malindretos et al., 2007^51^ | Patients on hemodialysis  (*n* = 20) | Patients received one session of hemodialysis without i.v. iron and thereafter two i.v. iron infusions at two different hemodialysis sessions: slow (60 min) infusion of 100 mg iron sucrose and 100 mg iron dextran. | Hemodialysis with or without iron infusions: ↔︎ Oxidized LDL |
| Maruyama et al., 2007^52^ | Patients on hemodialysis  (*n* = 27) | Patients with hematocrit <30% and ferritin <100n g/mL received 40mg i.v. ferric saccharate after each hemodialysis session until hematocrit increased by 5%. | Repeated i.v. iron administration: ↑ 8-OHdG (ferritin levels stayed between 100 and 200) |
| Pai et al.,  2007^53^ | Patients on hemodialysis  (*n* = 12) | 100 mg of i.v. iron dextran, sodium ferric gluconate, and iron sucrose in random sequence, with a 2-week washout period between treatments. | I.v. iron administration: ↑ NTBI (with sodium ferric gluconate and iron sucrose formulations)  ↑ MDA (mostly with sodium ferric gluconate) ↑ HO-1 RNA (no differences among iron formulations) ↑ Antibodies to oxidized LDL (only with iron dextran formulation) |
| Saglam et al.,  2007^54^ | Patients on continuous peritoneal dialysis (*n* = 12) | 100 mg i.v. iron sucrose administered over 30 min | Iron supplementation: ↔︎ Erythrocyte SOD, CAT, GPx, MDA activities  Only in patients with ferritin >400 ng/mL the MDA levels significantly increased 60 min after iron infusion. |
| Anraku et al.,  2008^55^ | Patients on hemodialysis  (*n* = 22) | 1. 40 mg i.v. chondroitin sulfate–iron colloid 3x/week for four weeks (520 mg total iron) 2. Weekly 40 mg i.v. iron for three months (520 mg total iron) | Compared with weekly iron infusions, iron supplementation thrice a week:  ↑ AOPP and oxidation of serum albumin |
| Kuo et al.,  2008^56^ | Patients on hemodialysis  (*n* = 110) | Phase I: Patients received single dose of either 20 mg, 50 mg, 100 mg, 200 mg, or 500 mg i.v. iron sucrose.  After 4 weeks, phase II: 1. Weekly 100 mg i.v. iron sucrose 2. Placebo | Single i.v. iron sucrose dose: ↑ Lymphocyte 8-OHdG (only in doses ≥200 mg)  Weekly i.v. iron supplementation: ↑ Lymphocyte 8-OHdG, ROS ↑ GSSG:GSH ratio ↓ ascorbate, ⍺-tocopherol |
| Stefánsson et al., 2008^57^ | Patients on hemodialysis  (*n* = 20) | 100 mg i.v. iron saccharate administered as bolus over 5 min | Iron supplementation: ↑ NTBI, MPO, protein carbonyl, BDC-LDL |
| Van Campenhout  et al., 2008^58^ | Patients on hemodialysis  (*n* = 11) | 100 mg i.v. iron saccharate administered over 30 min at the end of hemodialysis | Iron supplementation:  ↑ NTBI, MDA, thiols ↓ TEAC |
| Rangel et al.,  2010^59^ | Patients on hemodialysis  (*n* = 26) | 1. Repeated i.v. iron saccharate infusions during hemodialysis 2. Control group | I.v. iron supplementation induces transient increase in LPI (labile plasma iron). |
| Pai et al.,  2011^60^ | Patients on hemodialysis  (*n* = 10)  Healthy volunteers  (*n* = 4) | 1. 100 mg i.v. iron sucrose 2. 100 mg i.v. iron dextran Washout period — 2 weeks | Iron supplementation in patients on hemodialysis: ↑ NTBI (especially after iron sucrose administration)  ↑ Intracellular ROS generation ↓ Mitochondrial membrane potential ↔︎ F_2_-isoprostane  Iron supplementation in healthy volunteers: ↑ NTBI ↓ Mitochondrial membrane potential ↔︎ F_2_-isoprostane |
| Stefánsson et al., 2011^61^ | Patients on hemodialysis  (*n* = 20) | 1. 100 mg i.v. iron sucrose 2. 100 mg i.v. iron dextran Washout period— 4 weeks | I.v. iron supplementation: ↑ NTBI ↑ AFR and protein carbonyl (after iron sucrose administration)  ↔︎ Ox-LDL, TEAC |
| Conner et al.,  2012^62^ | Patients on hemodialysis  (*n* = 13) Healthy volunteers  (*n* = 4) | 1. 100 mg i.v. iron sucrose 2. 100 mg i.v. iron sucrose + 300 mg i.v. vitamin C  Washout period — 2 weeks | Iron supplementation: ↑ F_2_-isoprostane (especially with vitamin C co-administration)  ↑ NTBI, intracellular ROS generation ↓ Mitochondrial membrane potential |
| Martin-Malo et al., 2012^63^ | Patients on hemodialysis  (*n* = 10) | 1. Placebo (saline infusion) 2. 62.5 mg i.v. ferric gluconate 3. 100 mg i.v. iron dextran 4. 100 mg i.v. iron sucrose 5. 100 mg i.v. ferric carboxymaltose  Washout period — 2 weeks | Iron supplementation: ↑ The percentage of mononuclear cells with ROS production, ICAM-1, and apoptosis  No significant differences between different iron preparations. |
| Dogaru et al.,  2015^64^ | Patients on hemodialysis  (*n* = 20) | 1. No iron supplementation 2. 100 mg iron sucrose via venous line 3. 100 mg iron sucrose via the arterial extracorporeal circulation One session each with 1 week washout period. | Iron supplementation: ↔︎ TAC in erythrocytes  Compared with arterial extracorporeal administration, venous iron administration: ↑ GPx activity in erythrocytes |
| Nakayama et al., 2018^65^ | Patients on hemodialysis  (*n* = 6) | 1. No supplementation 2. 480 mg iron as oral ferric citrate hydrate  3. 40 mg iron as i.v. saccharated ferric oxide Washout period — 1 week | I.v. iron supplementation: ↑ NTBI, MPO ↓ Thioredoxin ↔︎ 8-OHdG, TBARS, d-ROM, BAP  Oral iron supplementation: ↔︎ NTBI, MPO, thioredoxin, d-ROM, BAP |
| Reggiani et al.,  2022^66^ | Patients on hemodialysis  (*n* = 24) | Patients switched from weekly 62.5 mg i.v. ferric gluconate to weekly 90 mg oral sucrosomial iron for 3 months | Switch to oral sucrosomial iron from i.v. iron supplementation: ↓ Protein carbonyls, di-tyrosines, AOPPs (non-significant decrease) |

*CRD: Carbonyl reactive derivatives; MDA: malondialdehyde; SOD: superoxide dismutase; GSH: glutathione; GSSH:* *oxidized glutathione; GPx: glutathione peroxidase; GSH-Rd: glutathione reductase; TAC: total antioxidant capacity; TOC: total oxidant capacity; FRAP: Ferric reducing/antioxidant power of plasma; TBARS : thiobarbituric acid reactive substances; 8-OHdG: 8-hydroxy-20-deoxyguanosin; AFR: ascorbyl free radical; NTBI: non-transferrin-bound-iron; TEAC: Trolox equivalent antioxidant capacity; LPO: lipid peroxide levels; LOOH: lipid hydroperoxide; CTCMC: Carotino® Tocotrienol Carotene Mixed Concentrate; i.m.: medication administered intramuscularly; i.v.: medication administered intravenously; AOPP: advanced oxidation protein products; MPO: myeloperoxidase; 8-ISO: 8α-isoprostanes; d-ROM: Reactive Oxygen Metabolites; hsCRP: highly-sensitive C-reactive Protein; IL: interleukin; BAP: biological antioxidant potential; TNF-α: Tumor necrosis factor alpha; FRAP: ferric reducing ability of plasma; ICAM-1: Inter-Cellular Adhesion Molecule-1; PAPP-A: Pregnancy-associated plasma protein-A; BDC-LDL: baseline diene conjugation in low-density lipoproteins; LPI: labile plasma iron.*

**Supplementary table S3: High-level summary of findings regarding the effect of iron supplementation on intestinal microbiota in infants and toddlers.**

| **Author** | **Population** | **Intervention** | **Effect of intervention on intestinal inflammation and microbiota** |
| --- | --- | --- | --- |
| Krebs et al., 2013^67^ | Exclusively breastfed 5-month-old infants  (*n* = 45) | Randomized to primary complementary food through 6–9 months of age: 1. Commercially available pureed meats 2. Iron and zinc fortified cereals  3. Iron-only fortified cereals | Compared with iron- and zinc-fortified cereals and meat purees, iron-only fortified cereal supplementation: ↓ phylum Actinobacteria (specifically genera Bifidobacterium and Rothia)  ↓ phylum Firmicutes (specifically Lactobacillales genus)  ↑ phylum Bacteroidetes (specifically Bacteroidales genus)  Clostridium Group XIVa increased by 40% in the meat feeding group, but only 10% in other groups. |
| Jaeggi et al., 2015^68^ | 6-month-old Kenyan infants (*n* = 115) | Randomized to consume fortified maize porridge daily for 4 months: 1. MNP with 2.5 mg iron as NaFeEDTA (+2.5mgFeMNP)  2. MNP without iron (-2.5mgFeMNP)  3. MNP with 12.5 mg iron as ferrous fumarate (+12.5mgFeMNP) 4. MNP without iron (-12.5mgFeMNP) | High prevalence of pathogens (including Salmonella Clostridium difficile, Clostridium perfringens, and pathogenic Escherichia coli) at baseline.  Iron-fortified MNPs: ↑ family Enterobacteria (genus Escherichia/Shigella, also pathogenic Escherichia coli strains)  ↑ Enterobacteria/Bifidobacteria ratio  ↑ phylum Firmicutes  ↑ FCP (only in +12.5mgFeMNP and iron-sufficient groups)  ↔︎ Fecal acetate, propionate, or butyrate concentrations |
| Cheung et al., 2016^69^ | 6-month-old Malawian infants  (*n* = 213) | Randomized to receive supplementary feeding for 12 months: 1. No supplementary feeding (control group). 2. Fortified LNS containing 6 mg Fe/dose. Milk-protein base. 3. Fortified LNS containing 6 mg Fe/dose. Soya-protein base.  4. Fortified corn–soya blend containing 5.46 mg Fe/dose. | Nutritional supplementation by LNS or corn–soya blends did not affect the gut microbiota profile. |
| Tang et al., 2016^70^ | 9 to 24-month-old infants and toddlers with ID or IDA (*n* = 36) | Randomized to receive for 8 weeks: 1. Iron (6 mg/kg/d) + placebo  2. Iron (6 mg/kg/d) + vitamin E (18 mg/d) | Iron with or without vitamin E supplementation: ↑ ⍺-diversity  ↓ genus Escherichia  ↔︎ FCP, TNF-⍺, IL-4  Compared with iron-only supplementation, iron + vitamin E supplementation:  ↑ genus Roseburia  ↑ family Lachnospiraceae  ↓ family Bacteroidaceae |
| Aakko et al., 2017^71^ | 5.5 to 6.5-month-old Malawian infants (*n* = 160) | Randomized to receive for 12 months: 1. No LNS (control group). 2. Fortified LNS containing 6mg Fe/dose. Milk-protein base. 3. Fortified LNS containing 6mg Fe/dose. Soya-protein base.  4. Fortified corn–soya blend containing 5.46 mg Fe/dose. | The study aimed to investigate the composition of gut microbiota, specifically Bifidobacterium and Staphylococcus aureus. The dietary supplementation did not influence the Bifidobacterium and Staphylococcus aureus microbiota composition of the study infants. |
| Paganini et al., 2017^72^ | 6.5 to 9.5-month-old Kenyan infants  (*n* = 155) | Randomized to receive for 4 months: 1. MNP without iron (control group). 2. MNP with 5 mg iron (2.5 mg as NaFeEDTA and 2.5 mg as ferrous fumarate). FeMNP group. 3. MNP as the Fe group but with 7.5 g GOS (FeGOS group). | Compared with the control and FeGOS groups, supplementation with FeMNP: ↓ genera Bifidobacterium and Lactobacillus  ↑ order Clostridiales  ↑ families Ruminococcaceae, Lachnospiraceae, and Erysipelotrichaceae  ↑ abundances of virulence and toxin genes of pathogens  ↑ plasma I-FABP  ↑ incidence of treated RTIs  No differences were seen in FCP between groups. |
| Tang et al., 2017^73^ | 6-months-old Kenyan infants with or without mild anemia within malaria-endemic area  (*n* = 33) | Randomized to receive for 3 months: 1. MNP containing 12.5 mg iron (MNP+Fe) 2. MNP containing no iron (MNP−Fe) 3. Placebo (control group) | Control group: ↓ IL-8  ↓ genus Escherichia/Shigella  ↓ genus Bifidobacterium  MNP-Fe group: ↑ genus Clostridium ↓ genus Escherichia/Shigella  ↔︎ inflammatory markers  MNP+Fe group: ↓ genus Bifidobacterium ↔︎ inflammatory markers |
| Paganini et al., 2019^74^ | 8 to 10-month-old infants (*n* = 28) | Randomized to receive: 1. antibiotics for 5 days and iron-MNPs for 40 days 5 mg/dose (Fe+ab+ group) 2. Antibiotics and no-iron-MNPs (Fe−ab+ group) 3. Iron-MNPs but no antibiotics (Fe+ab− group). Fe 5 mg/dose as 2.5 mg ferrous fumarate and 2.5 mg NaFeEDTA. 4. No antibiotics and no iron-MNPs (Fe−ab− group). | Compared with Fe−ab+, in Fe+ab+ group: ↑ Enterobacteriaceae and Eubacteriaceae  ↑ Clostridium difficile  ↓ genus Bifidobacterium  ↔︎ Escherichia coli  ↔︎ VTGs of pathogenic Escherichia coli |
| Simonytė Sjödin et al., 2019^75^ | 6-month-old Swedish infants without iron deficiency  (*n* = 53) | Randomized to receive for 45 days: 1. Low-iron-fortified formula with 1.2 mg Fe/day (low-Fe group) 2. High-iron-fortified formula with 6.6 mg Fe/day (high-Fe group) 3. Non-fortified formula with liquid ferrous sulfate supplementation with 6.6 mg Fe/day (Fe-drops group).  All formula had added galactooligosaccharides. | High-Fe group: ↓ genus Bifidobacterium  Compared with high-Fe group, the Fe-drops group: ↓ Lactobacillus sp.  ↓ genus Streptococcus  ↑ genera Clostridium and Bacteroides  Compared with low-Fe group, the high-Fe group: ↑ Lactobacillus sp.  No differences were seen in FCP between groups. |
| Kamng’ona et al., 2020^76^ | Pregnant women and their infants  (*n* = 869) | Randomized to receive: 1. MMN capsule daily during pregnancy and 6 months postpartum, which included 20 mg iron and 400 μg of folic acid (MMN group). MMN was not given to infants. 2. LNS sachet daily during pregnancy and 6 months postpartum. The LNS contained the same 18 micronutrients as the MMN capsule, with 4 additional minerals, protein and fat (LNS group). LNS was also given to infants after 6-months of age. 3. Patients in this group received 60 mg iron and 400 μg of folic acid in an IFA capsule each day until delivery, followed by a placebo capsule (low dose calcium) until 6 months postpartum (IFA group). IFA was not given to infants. | There were no differences in infants’ microbiota maturity and α-diversity between IFA and MMN groups; these groups were combined (IFA + MMN).  Mean α diversity was higher in the LNS group at 18 months, but no significant differences were observed at 1, 6, 12, or 30 months or in microbiota maturity compared with the IFA+MMN group. |
| Owolabi et al., 2021^77^ | 12- to 36-month-old malnourished Nigerian toddlers with anemia  (*n* = 184) | Randomized to receive for 6 months: 1. 200 mL multi-nutrient fortified dairy-based drink (2.24 mg ferrous sulfate per day). 2. 400 mL multi-nutrient fortified dairy-based drink (4.48 mg ferrous sulfate per day). 3. 600 mL multi-nutrient fortified dairy-based drink (6.72 mg ferrous sulfate per day). | All groups: ↓ Enterobacteriaceae  ↔︎ Bifidobacteriaceae  ↔︎ pathogenic Escherchicia coli  CRP decreased in the 600mL group; no differences were seen in the other groups. |
| Popovic et al., 2021^78^ | 6-month-old infants from Pakistan (*n* = 80) | Randomized to receive supplementation for 12 months:  1. No MNP (control group) 2. MNP with 12.5 mg ferrous fumarate (Fe-group) 3. MNP with 12.5 mg ferrous fumarate and 10mg zinc gluconate (Fe-Zn group) | At 24-month age, Fe-group compared with control and Fe-Zn group: ↑ genus Escherichia/Shigella  ↑ carriage of protozoa and mucormycetes  ↓ Bifidobacterium ↔︎ Firmicutes  In Fe-Zn group rather than Fe-group, Firmicutes were decreased. |
| De Goffau et al., 2022^79^ | 7- to 37-month old Gambian infants  (*n* = 616) | Randomized to receive: 1. 20 mg/day IHAT (IHAT group) 2. 12.5 mg/day ferrous sulfate (FeSO_4_ group) 3. Saccharose powder without iron (control group) | The combined time point analysis showed a positive association between Megamonas funiformis and iron supplementation, yet there was no association when analyzing day 1, day 15 or day 85 samples separately. |

*ID: iron deficiency, IDA: iron deficiency anemia, FCP: fecal calprotectin,* NaFeEDTA: sodium ferric ethylenediaminetetraacetate, LNS: lipid-based nutritional supplement, TNF-⍺: tumor necrosis factor alpha, IL-4: interleukin 4, GOS: galactooligosaccharide, I-FAB: plasma intestinal fatty acid-binding protein, RTI: respiratory tract infection. VTG: virulence and toxin genes, MMN: multiple micronutrients, IFA: iron and folic acid, IHAT: iron hydroxide adipate tartrate.

**Supplementary table S4: High-level summary of findings regarding the effect of iron supplementation on intestinal microbiota in children.**

| **Author** | **Population** | **Intervention** | **Effect of intervention on intestinal inflammation and microbiota** |
| --- | --- | --- | --- |
| Zimmermann et al., 2010^80^ | 6- to 14-year-old children living in Cote d’Ivoire (*n* = 139) | Randomized to receive for 6 months:  1. Iron-fortified biscuits containing 20 mg as electrolytic iron, 4x/week 2. Non-fortified biscuits 4x/week | Iron fortification: ↑ FCP  ↑ Enterobacteria  ↓ Lactobacilli  ↔︎ Bifidobacteria and Bacteroides |
| Dostal et al., 2014^81^ | 6- to 11-year-old children living in South Africa (*n* = 73) | Randomized to reveid for 38 weeks: 1. 50 mg FeSO4, 4x/week together with a vitamin C-enriched beverage (10 mg/serving) 2. Placebo tablet together with a vitamin C-enriched beverage (10 mg/serving) 3. Iron-sufficient children were included but not treated as a reference group | No significant effects of iron supplementation or time-supplementation interaction on the concentrations of bacterial groups, fecal SCFA, or FCP were observed between the groups. |
| Rahman et al., 2021^82^ | 2- to 5-year-old Bangladeshi children from area with a high groundwater iron concentration: ≥ 2 mg/L (*n* = 100) | Randomized to receive for 2 months: 1. MNP with 12.5 mg ferrous fumarate (high-iron MNP group) 2. MNP with 5 mg ferrous fumarate (low-iron MNP group) | A low-iron MNP supplementation did not have a significant impact on gut microbiota composition or diversity compared to the standard MNP.  However, Bifidobacterium and Lactobacillus were negatively associated with iron concentration of tube-well water. |
| Goosen et al., 2022^83^ | 8- to 13-year-old children with suppressed HIV (< 50 HIV RNA copies/mL) and without HIV (*n* = 64) | All participants had iron deficiency and were treated with 55 mg od ferrous sulfate for 3 months. | No significant effects of iron supplementation on ⍺-diversity, relative abundances of intestinal bacteria, or FCP in children with and without HIV.  However, intestinal microbiota in children with HIV differed from children without HIV. |

*FCP: fecal calprotectin, SCFA: short-chain fatty acids, HIV: human immunodeficiency virus, MNP: micronutrient powder.*

**Supplementary table S5: High-level summary of findings regarding the effect of iron supplementation on intestinal microbiota in adults.**

| **Author** | **Population** | **Intervention** | **Effect of intervention on intestinal inflammation and microbiota** |
| --- | --- | --- | --- |
| Lee et al., 2017^84^ | Adults with IBD (*n* = 53) Patients with ID not associated with inflammation (*n* = 19) | Randomized to receive for 3 months: 1. 300 mg bd oral iron sulfate 2. 300 mg intravenous iron sucrose (max. 4 times) | Iron supplementation had no effect on disease activity scores or inflammatory parameters.  Overtime oral or intravenous iron supplementation did not overrule IBD-specific clustering (e.g., ↓ proportions of Clostridiales).  Compared with intravenous iron, oral iron supplementation:  ↑ Bifidobacterium  ↓ Faecalibacterium prausnitzii, Ruminococcus bromii, Dorea sp., and Collinsella aerofaciens |
| Dekker Nitert et al., 2018^85^ | Obese or overweight pregnant women  (*n* = 159) | Pregnant women were consuming: 1. Low-iron supplementation (<60 mg/d). Supplements contained either ferrous sulfate or ferrous glycinate. 2. High-iron supplementation (≥60 mg/d). All supplements contained ferrous sulfate. | Iron supplementation did not significantly affect the composition of intestinal microbiota.  Ruminococcus, Suterella, Lachnospira, and Lactobacillus were more abundant in low-iron group, which was also associated with butanoate metabolism and lipid biosynthesis proteins.  Acidaminococcus was overrepresented in high iron groups and was associated with amino acid metabolism and siderophore bioshynthesis. |
| Ahmed et al., 2020^86^ | Patients with IDA  (*n* = 45) | 200 mg oral ferrous sulfate, max. 3 times a day. | Paired sample analysis revealed no significant differences.  Unpaired sample analysis showed that iron supplementation:  ↑ Octanal, heptanal  ↓ Ethyl hexanoate and 2,4-dimethylpentan-3-ol |
| Iguchi et al., 2020^87^ | Patients on maintenance hemodialysis (*n* = 38) | For 12 weeks patients received: 1. SFO (750 mg with each meal) in addition to or in place of other phosphate binders (SFO group) 2. Regular phosphate binders, no SFO (control group) | SFO:  ↔︎ diversity and major components in phylum, class, order, family, gene, and species |
| Mahalhal et al., 2021^88^ | Animal experiment:  Wild-type C57BL/six female mice, aged 8–9 weeks old with DSS-induced acute colitis  (*n* = 40)  Human study: Patients with IDA  (*n* = 10)  Patients with IBD and IDA (*n* =6) | Groups of mice were fed and euthanized on day 10: 1. Standard chow containing 200 ppm iron (SC group) 2. SC with 200 ppm ferrous sulfate supplementation (FS group) 3. SC with 200 ppm ferric maltol supplementation (FM group)  1. Patients with IDA were treated with 200 mg bd ferrous sulfate for 4 weeks 2. Patients with quiescent IBD and IDA received 30 mg bd ferric maltol for 4 weeks | In mice, the severity of DSS-induced colitis was greater in FS than SC and FM groups. After DSS treatment, there was a decrease in many genera in the SC and FS groups, whereas Lactobacillales increased in mice that received FMS.  In humans, ferrous sulfate supplementation:  ↓ genera Dorea and Turicibacter  ↑ genera Butyrivibrio, Megamonas, Megasphaera, Lactobacillus, Acidaminococcus  Ferric maltol was not associated with any measurable change.  ⍺-diversity was not different between the groups, whereas β-diversity analysis showed that the main factor influencing the variability was iron deficiency and the diagnosis (IBD vs. non-IBD). |
| Phipps et al., 2021^89^ | Patients with non-metastasized colorectal adenocarcinoma and anemia (*n* = 40) | Randomized to: 1. 200 mg bd oral ferrous sulfate 2. Intravenous ferric carboxymaltose. Dosed according to weight and hemoglobin level. | In paired analysis of patients treated with oral iron, the off-tumor microbiota was enriched with Bacteroidaceae family and Bacteroides genus; the on-tumor microbiota showed a greater abundance of Nocardiaceae, Intrasporangiaceae, and Brevibacteriaceae families and Prevotella, Nocardioides, Kocuria, Brevibacterium, Veillonella and Catenibacterium genera.  In paired analysis of patients treated with intravenous iron, the off-tumor microbiota was enriched with Firmicutes phylum and the Clostridia spp. along with higher abundances of the Clostridiales and Sphingomonadales orders, the Sphingomonadaceae family and the Paraprevotella genus; the on-tumor microbiota showed higher abundance of Epsilonbacteraeota phylum, Campylobacteria class, Campylobacteriales order, Campylobacteraceae, Propionibacteriaceae and Porphyro-monadaceae families and Campylobacter, Porphyromonas and Cutibacterium. |
| Abdelbary et al., 2022^90^ | Patients on hemodialysis  (*n* = 11) | Patients received 500 mg SFO with each meal (1.5 g ferric iron intake a day). | The significant changes observed in fecal samples were:  ↑ Veillonella sp. and Ruminococcus torques group  ↓ Subdoligranulum |
| Liu et al., 2023^91^ | Patients with IDA on maintenance hemodialysis (*n* = 28) | Randomized to receive: 1. 200 mg od oral ferrous succinate for at least 2 months 2. 100 mg intravenous iron sucrose, thrice weekly, infused at least 10 times | Lactobacillus and Vagococcus sp were found in higher abundances in patients treated with oral iron than intravenous iron.  Oral iron:  ↑ Bacteroidetes phylum, Bacteroides genus  ↓ Firmicutes phylum, Blautia and Coprococcus genera  ↓ ⍺-diversity  IV group abundance:  ↑ Verucomicrobiae class  ↑ Orders Verrucomicrobiales, Actinomycetales, Oceanospirillales, Rhizobiales, and Gemellales  ↑ Families Enterococcaceae, Peptostreptococcaceae, Verrucomicrobiaceae, Leuconostoaceae, Micrococcaceae, Camobateriaceae, Halomonadaceae, Actinomycetaceae, Hyphomicrobiaceae, and Gemellaceae  ↑ Genera Enterococcus, Ruminococcus, VadinHB04, Akkermansia, Chelativorans, Rothia, Halo-monas, Actinomyces, and Devosia |
| Seo et al., 2023^92^ | Premenopausal women with and without IDA (*n* = 31) | Patients with IDA received either 1.0–1.5 g intravenous iron sucrose or 80 mg bd oral iron sulfate for 3–6 months. | Iron supplementation:  ↓ Betoproteobacteria class  ↓ Erysipelotrichi class–Erysipelotrichales order–Erysipelotrichaceae family lineage  Compared with healthy women, women with with IDA had:  ↑ Negativicutes class  ↓ Clostridia class–Clostridiales order–Ruminococcaceae family–Faecalibacterium genus lineage  Compared with healthy women, women treated with iron supplementation had:  ↑ Veilonella genus  ↓ Coriobacteriia class–Coriobacteriales order–Coriobacteriaceae family–Collinsell genus lineage |

*UC: ulcerative colitis, CD: Crohn’s disease, IBD: inflammatory bowel disease, ID: iron deficiency, IDA: iron deficiency anemia, SFO: sucroferric oxyhydroxide, IS: indoxyl sulfate, PCS: p-cresyl sulfate, DSS: dextran sodium sulfate, FCP: fecal calprotectin, Bd: twice a day, od: once a day.*

**REFERENCES**

1. Rooyakkers TM, Stroes ES, Kooistra MP, et al. Ferric saccharate induces oxygen radical stress and endothelial dysfunction in vivo. Eur J Clin Invest 2002;32 Suppl 1:9-16.

2. Schümann K, Kroll S, Weiss G, et al. Monitoring of hematological, inflammatory and oxidative reactions to acute oral iron exposure in human volunteers: preliminary screening for selection of potentially-responsive biomarkers. Toxicology 2005;212:10-23.

3. Colpo E, de Bem AF, Pieniz S, et al. A single high dose of ascorbic acid and iron is not correlated with oxidative stress in healthy volunteers. Ann Nutr Metab 2008;53:79-85.

4. Orozco MN, Arriaga C, Solomons NW, et al. Equivalent effects on fecal reactive oxygen species generation with oral supplementation of three iron compounds: ferrous sulfate, sodium iron EDTA and iron polymaltose. Ann Nutr Metab 2012;60:108-14.

5. Orozco MN, Solomons NW, Schümann K, et al. Response of urinary biomarkers of systemic oxidation to oral iron supplementation in healthy men. Food Nutr Bull 2012;33:53-62.

6. Wang J, Radics G, Whelehan M, et al. Novel Iron-Whey Protein Microspheres Protect Gut Epithelial Cells from Iron-Related Oxidative Stress and Damage and Improve Iron Absorption in Fasting Adults. Acta Haematol 2017;138:223-232.

7. Abiri B, Vafa M, Azizi-Soleiman F, et al. Changes in Bone Turnover, Inflammatory, Oxidative Stress, and Metabolic Markers in Women Consuming Iron plus Vitamin D Supplements: a Randomized Clinical Trial. Biol Trace Elem Res 2021;199:2590-2601.

8. Erichsen K, Hausken T, Ulvik RJ, et al. Ferrous fumarate deteriorated plasma antioxidant status in patients with Crohn disease. Scand J Gastroenterol 2003;38:543-8.

9. Erichsen K, Ulvik RJ, Grimstad T, et al. Effects of ferrous sulphate and non-ionic iron-polymaltose complex on markers of oxidative tissue damage in patients with inflammatory bowel disease. Aliment Pharmacol Ther 2005;22:831-8.

10. Erichsen K, Ulvik RJ, Nysaeter G, et al. Oral ferrous fumarate or intravenous iron sucrose for patients with inflammatory bowel disease. Scand J Gastroenterol 2005;40:1058-65.

11. Lasocki S, Piednoir P, Couffignal C, et al. Does IV Iron Induce Plasma Oxidative Stress in Critically Ill Patients? A Comparison With Healthy Volunteers. Crit Care Med 2016;44:521-30.

12. Pérez-Peiró M, Martín-Ontiyuelo C, Rodó-Pi A, et al. Iron Replacement and Redox Balance in Non-Anemic and Mildly Anemic Iron Deficiency COPD Patients: Insights from a Clinical Trial. Biomedicines 2021;9.

13. Isler M, Delibas N, Guclu M, et al. Superoxide dismutase and glutathione peroxidase in erythrocytes of patients with iron deficiency anemia: effects of different treatment modalities. Croat Med J 2002;43:16-9.

14. Kurtoglu E, Ugur A, Baltaci AK, et al. Effect of iron supplementation on oxidative stress and antioxidant status in iron-deficiency anemia. Biol Trace Elem Res 2003;96:117-23.

15. Binkoski AE, Kris-Etherton PM, Beard JL. Iron supplementation does not affect the susceptibility of LDL to oxidative modification in women with low iron status. J Nutr 2004;134:99-103.

16. Ruivard M, Feillet-Coudray C, Rambeau M, et al. Effect of daily versus twice weekly long-term iron supplementation on iron absorption and status in iron-deficient women: a stable isotope study. Clin Biochem 2006;39:700-7.

17. Sundaram RC, Selvaraj N, Vijayan G, et al. Increased plasma malondialdehyde and fructosamine in iron deficiency anemia: effect of treatment. Biomed Pharmacother 2007;61:682-5.

18. King SM, Donangelo CM, Knutson MD, et al. Daily supplementation with iron increases lipid peroxidation in young women with low iron stores. Exp Biol Med (Maywood) 2008;233:701-7.

19. Tiwari AK, Mahdi AA, Chandyan S, et al. Oral iron supplementation leads to oxidative imbalance in anemic women: a prospective study. Clin Nutr 2011;30:188-93.

20. Akarsu S, Demir H, Selek S, et al. Iron deficiency anemia and levels of oxidative stress induced by treatment modality. Pediatr Int 2013;55:289-95.

21. Khoshfetrat MR, Mohammadi F, Mortazavi S, et al. The effect of iron-vitamin C co-supplementation on biomarkers of oxidative stress in iron-deficient female youth. Biol Trace Elem Res 2013;153:171-7.

22. Lachili B, Hininger I, Faure H, et al. Increased lipid peroxidation in pregnant women after iron and vitamin C supplementation. Biol Trace Elem Res 2001;83:103-10.

23. Rehema A, Zilmer K, Klaar U, et al. Ferrous iron administration during pregnancy and adaptational oxidative stress (Pilot study). Medicina (Kaunas) 2004;40:547-52.

24. Ma AG, Schouten EG, Sun YY, et al. Supplementation of iron alone and combined with vitamins improves haematological status, erythrocyte membrane fluidity and oxidative stress in anaemic pregnant women. Br J Nutr 2010;104:1655-61.

25. Han XX, Sun YY, Ma AG, et al. Moderate NaFeEDTA and ferrous sulfate supplementation can improve both hematologic status and oxidative stress in anemic pregnant women. Asia Pac J Clin Nutr 2011;20:514-20.

26. Viteri FE, Casanueva E, Tolentino MC, et al. Antenatal iron supplements consumed daily produce oxidative stress in contrast to weekly supplementation in Mexican non-anemic women. Reprod Toxicol 2012;34:125-32.

27. Korkmaz V, Ozkaya E, Seven BY, et al. Comparison of oxidative stress in pregnancies with and without first trimester iron supplement: a randomized double-blind controlled trial. J Matern Fetal Neonatal Med 2014;27:1535-8.

28. Lymperaki E, Tsikopoulos A, Makedou K, et al. Impact of iron and folic acid supplementation on oxidative stress during pregnancy. J Obstet Gynaecol 2015;35:803-6.

29. Jorgensen JM, Yang Z, Lönnerdal B, et al. Effect of iron supplementation during lactation on maternal iron status and oxidative stress: A randomized controlled trial. Matern Child Nutr 2017;13.

30. Jacob OM, Kant S, Haldar P, et al. Intravenous Iron sucrose and change in hemoglobin, ferritin, and oxidative stress markers among moderately anemic pregnant women attending a secondary care level Hospital in Northern India. Indian J Public Health 2020;64:11-16.

31. Agarwal R, Vasavada N, Sachs NG, et al. Oxidative stress and renal injury with intravenous iron in patients with chronic kidney disease. Kidney Int 2004;65:2279-89.

32. Leehey DJ, Palubiak DJ, Chebrolu S, et al. Sodium ferric gluconate causes oxidative stress but not acute renal injury in patients with chronic kidney disease: a pilot study. Nephrol Dial Transplant 2005;20:135-40.

33. Sezer MT, Akin H, Demir M, et al. The effect of serum albumin level on iron-induced oxidative stress in chronic renal failure patients. J Nephrol 2007;20:196-203.

34. Ganguli A, Kohli HS, Khullar M, et al. Lipid peroxidation products formation with various intravenous iron preparations in chronic kidney disease. Ren Fail 2009;31:106-10.

35. Kassianides X, Gordon A, Sturmey R, et al. The comparative effects of intravenous iron on oxidative stress and inflammation in patients with chronic kidney disease and iron deficiency: a randomized controlled pilot study. Kidney Res Clin Pract 2021;40:89-98.

36. Kassianides X, Allgar V, Macdougall IC, et al. Analysis of oxidative stress, inflammation and endothelial function following intravenous iron in chronic kidney disease in the Iron and Heart Trial. Sci Rep 2022;12:6853.

37. Kassianides X, White S, Bhandari S. Markers of Oxidative Stress, Inflammation and Endothelial Function following High-Dose Intravenous Iron in Patients with Non-Dialysis-Dependent Chronic Kidney Disease-A Pooled Analysis. Int J Mol Sci 2022;23.

38. Roob JM, Khoschsorur G, Tiran A, et al. Vitamin E attenuates oxidative stress induced by intravenous iron in patients on hemodialysis. J Am Soc Nephrol 2000;11:539-549.

39. Herrera J, Nava M, Romero F, et al. Melatonin prevents oxidative stress resulting from iron and erythropoietin administration. Am J Kidney Dis 2001;37:750-7.

40. Salahudeen AK, Oliver B, Bower JD, et al. Increase in plasma esterified F2-isoprostanes following intravenous iron infusion in patients on hemodialysis. Kidney Int 2001;60:1525-31.

41. Tovbin D, Mazor D, Vorobiov M, et al. Induction of protein oxidation by intravenous iron in hemodialysis patients: role of inflammation. Am J Kidney Dis 2002;40:1005-12.

42. Cavdar C, Temiz A, Yeniçerioğlu Y, et al. The effects of intravenous iron treatment on oxidant stress and erythrocyte deformability in hemodialysis patients. Scand J Urol Nephrol 2003;37:77-82.

43. Michelis R, Gery R, Sela S, et al. Carbonyl stress induced by intravenous iron during haemodialysis. Nephrol Dial Transplant 2003;18:924-30.

44. Anraku M, Kitamura K, Shinohara A, et al. Intravenous iron administration induces oxidation of serum albumin in hemodialysis patients. Kidney Int 2004;66:841-8.

45. Tiranathanagul K, Eiam-Ong S, Tosukhowong P, et al. Oxidative stress from rapid versus slow intravenous iron replacement in haemodialysis patients. Nephrology (Carlton) 2004;9:217-22.

46. Driss F, Vrtovsnik F, Katsahian S, et al. Effects of intravenous polymaltose iron on oxidant stress and non-transferrin-bound iron in hemodialysis patients. Nephron Clin Pract 2005;99:c63-7.

47. Hodkova M, Kalousova M, Dusilova-Sulkova S, et al. Intravenous iron gluconate administration increases circulating PAPP-A in hemodialysis patients. Ren Fail 2005;27:707-11.

48. Mimić-Oka J, Savić-Radojević A, Pljesa-Ercegovac M, et al. Evaluation of oxidative stress after repeated intravenous iron supplementation. Ren Fail 2005;27:345-51.

49. Eiselt J, Racek J, Opatrný K, Jr., et al. The effect of intravenous iron on oxidative stress in hemodialysis patients at various levels of vitamin C. Blood Purif 2006;24:531-7.

50. de Vecchi AF, Novembrino C, Lonati S, et al. Two different modalities of iron gluconate i.v. administration: effects on iron, oxidative and inflammatory status in peritoneal dialysis patients. Nephrol Dial Transplant 2007;22:1709-13.

51. Malindretos P, Sarafidis PA, Rudenco I, et al. Slow intravenous iron administration does not aggravate oxidative stress and inflammatory biomarkers during hemodialysis: a comparative study between iron sucrose and iron dextran. Am J Nephrol 2007;27:572-9.

52. Maruyama Y, Nakayama M, Yoshimura K, et al. Effect of repeated intravenous iron administration in haemodialysis patients on serum 8-hydroxy-2'-deoxyguanosine levels. Nephrol Dial Transplant 2007;22:1407-12.

53. Pai AB, Boyd AV, McQuade CR, et al. Comparison of oxidative stress markers after intravenous administration of iron dextran, sodium ferric gluconate, and iron sucrose in patients undergoing hemodialysis. Pharmacotherapy 2007;27:343-50.

54. Saglam F, Cavdar C, Uysal S, et al. Effect of intravenous iron sucrose on oxidative stress in peritoneal dialysis patients. Ren Fail 2007;29:849-54.

55. Anraku M, Kitamura K, Shintomo R, et al. Effect of intravenous iron administration frequency on AOPP and inflammatory biomarkers in chronic hemodialysis patients: a pilot study. Clin Biochem 2008;41:1168-74.

56. Kuo KL, Hung SC, Wei YH, et al. Intravenous iron exacerbates oxidative DNA damage in peripheral blood lymphocytes in chronic hemodialysis patients. J Am Soc Nephrol 2008;19:1817-26.

57. Stefánsson BV, Haraldsson B, Nilsson U. Ascorbyl free radical reflects catalytically active iron after intravenous iron saccharate injection. Free Radic Biol Med 2008;45:1302-7.

58. Van Campenhout A, Van Campenhout C, Lagrou A, et al. Iron-induced oxidative stress in haemodialysis patients: a pilot study on the impact of diabetes. Biometals 2008;21:159-70.

59. Rangel EB, Espósito BP, Carneiro FD, et al. Labile plasma iron generation after intravenous iron is time-dependent and transitory in patients undergoing chronic hemodialysis. Ther Apher Dial 2010;14:186-92.

60. Pai AB, Conner T, McQuade CR, et al. Non-transferrin bound iron, cytokine activation and intracellular reactive oxygen species generation in hemodialysis patients receiving intravenous iron dextran or iron sucrose. Biometals 2011;24:603-13.

61. Stefánsson BV, Haraldsson B, Nilsson U. Acute oxidative stress following intravenous iron injection in patients on chronic hemodialysis: a comparison of iron-sucrose and iron-dextran. Nephron Clin Pract 2011;118:c249-56.

62. Conner TA, McQuade C, Olp J, et al. Effect of intravenous vitamin C on cytokine activation and oxidative stress in end-stage renal disease patients receiving intravenous iron sucrose. Biometals 2012;25:961-9.

63. Martin-Malo A, Merino A, Carracedo J, et al. Effects of intravenous iron on mononuclear cells during the haemodialysis session. Nephrol Dial Transplant 2012;27:2465-71.

64. Dogaru CB, Capusa C, Gaman L, et al. Venous versus arterial iron administration in haemodialysis. Influence on erythrocytes antioxidant parameters. J Med Life 2015;8 Spec Issue:69-73.

65. Nakayama M, Tani Y, Zhu WJ, et al. Oral Ferric Citrate Hydrate Associated With Less Oxidative Stress Than Intravenous Saccharated Ferric Oxide. Kidney Int Rep 2018;3:364-373.

66. Reggiani F, Colombo G, Astori E, et al. Preliminary experience on the use of sucrosomial iron in hemodialysis: focus on safety, hemoglobin maintenance and oxidative stress. Int Urol Nephrol 2022;54:1145-1153.

67. Krebs NF, Sherlock LG, Westcott J, et al. Effects of different complementary feeding regimens on iron status and enteric microbiota in breastfed infants. J Pediatr 2013;163:416-23.

68. Jaeggi T, Kortman GA, Moretti D, et al. Iron fortification adversely affects the gut microbiome, increases pathogen abundance and induces intestinal inflammation in Kenyan infants. Gut 2015;64:731-42.

69. Cheung YB, Xu Y, Mangani C, et al. Gut microbiota in Malawian infants in a nutritional supplementation trial. Trop Med Int Health 2016;21:283-90.

70. Tang M, Frank DN, Sherlock L, et al. Effect of Vitamin E With Therapeutic Iron Supplementation on Iron Repletion and Gut Microbiome in US Iron Deficient Infants and Toddlers. J Pediatr Gastroenterol Nutr 2016;63:379-85.

71. Aakko J, Grześkowiak Ł, Asukas T, et al. Lipid-based Nutrient Supplements Do Not Affect Gut Bifidobacterium Microbiota in Malawian Infants: A Randomized Trial. J Pediatr Gastroenterol Nutr 2017;64:610-615.

72. Paganini D, Uyoga MA, Kortman GAM, et al. Prebiotic galacto-oligosaccharides mitigate the adverse effects of iron fortification on the gut microbiome: a randomised controlled study in Kenyan infants. Gut 2017;66:1956-1967.

73. Tang M, Frank DN, Hendricks AE, et al. Iron in Micronutrient Powder Promotes an Unfavorable Gut Microbiota in Kenyan Infants. Nutrients 2017;9.

74. Paganini D, Uyoga MA, Kortman GAM, et al. Iron-containing micronutrient powders modify the effect of oral antibiotics on the infant gut microbiome and increase post-antibiotic diarrhoea risk: a controlled study in Kenya. Gut 2019;68:645-653.

75. Simonyté Sjödin K, Domellöf M, Lagerqvist C, et al. Administration of ferrous sulfate drops has significant effects on the gut microbiota of iron-sufficient infants: a randomised controlled study. Gut 2019;68:2095-2097.

76. Kamng'ona AW, Young R, Arnold CD, et al. Provision of Lipid-Based Nutrient Supplements to Mothers During Pregnancy and 6 Months Postpartum and to Their Infants from 6 to 18 Months Promotes Infant Gut Microbiota Diversity at 18 Months of Age but Not Microbiota Maturation in a Rural Malawian Setting: Secondary Outcomes of a Randomized Trial. J Nutr 2020;150:918-928.

77. Owolabi AJ, Senbanjo IO, Oshikoya KA, et al. Multi-Nutrient Fortified Dairy-Based Drink Reduces Anaemia without Observed Adverse Effects on Gut Microbiota in Anaemic Malnourished Nigerian Toddlers: A Randomised Dose-Response Study. Nutrients 2021;13.

78. Popovic A, Bourdon C, Wang PW, et al. Micronutrient supplements can promote disruptive protozoan and fungal communities in the developing infant gut. Nat Commun 2021;12:6729.

79. de Goffau MC, Jallow AT, Sanyang C, et al. Gut microbiomes from Gambian infants reveal the development of a non-industrialized Prevotella-based trophic network. Nat Microbiol 2022;7:132-144.

80. Zimmermann MB, Chassard C, Rohner F, et al. The effects of iron fortification on the gut microbiota in African children: a randomized controlled trial in Cote d'Ivoire. Am J Clin Nutr 2010;92:1406-15.

81. Dostal A, Baumgartner J, Riesen N, et al. Effects of iron supplementation on dominant bacterial groups in the gut, faecal SCFA and gut inflammation: a randomised, placebo-controlled intervention trial in South African children. Br J Nutr 2014;112:547-56.

82. Rahman S, Kortman GAM, Boekhorst J, et al. Effect of low-iron micronutrient powder (MNP) on the composition of gut microbiota of Bangladeshi children in a high-iron groundwater setting: a randomized controlled trial. Eur J Nutr 2021;60:3423-3436.

83. Goosen C, Proost S, Tito RY, et al. The effect of oral iron supplementation on the gut microbiota, gut inflammation, and iron status in iron-depleted South African school-age children with virally suppressed HIV and without HIV. Eur J Nutr 2022;61:2067-2078.

84. Lee T, Clavel T, Smirnov K, et al. Oral versus intravenous iron replacement therapy distinctly alters the gut microbiota and metabolome in patients with IBD. Gut 2017;66:863-871.

85. Dekker Nitert M, Gomez-Arango LF, Barrett HL, et al. Iron supplementation has minor effects on gut microbiota composition in overweight and obese women in early pregnancy. Br J Nutr 2018;120:283-289.

86. Ahmed A, Slater R, Lewis S, et al. Using Volatile Organic Compounds to Investigate the Effect of Oral Iron Supplementation on the Human Intestinal Metabolome. Molecules 2020;25.

87. Iguchi A, Yamamoto S, Oda A, et al. Effect of sucroferric oxyhydroxide on gastrointestinal microbiome and uremic toxins in patients with chronic kidney disease undergoing hemodialysis. Clin Exp Nephrol 2020;24:725-733.

88. Mahalhal A, Frau A, Burkitt MD, et al. Oral Ferric Maltol Does Not Adversely Affect the Intestinal Microbiome of Patients or Mice, But Ferrous Sulphate Does. Nutrients 2021;13.

89. Phipps O, Al-Hassi HO, Quraishi MN, et al. Oral and Intravenous Iron Therapy Differentially Alter the On- and Off-Tumor Microbiota in Anemic Colorectal Cancer Patients. Cancers (Basel) 2021;13.

90. Abdelbary MMH, Kuppe C, Michael SS, et al. Impact of sucroferric oxyhydroxide on the oral and intestinal microbiome in hemodialysis patients. Sci Rep 2022;12:9614.

91. Liu H, Wu W, Luo Y. Oral and intravenous iron treatment alter the gut microbiome differentially in dialysis patients. Int Urol Nephrol 2023;55:759-767.

92. Seo H, Yoon SY, Ul-Haq A, et al. The Effects of Iron Deficiency on the Gut Microbiota in Women of Childbearing Age. Nutrients 2023;15.
